# Background segmentation in difficult weather conditions

**DOI:** 10.7717/peerj-cs.962

**Published:** 2022-05-13

**Authors:** Lukasz Karbowiak, Janusz Bobulski

**Affiliations:** Department of Computer Science, Czestochowa University of Technology, Czestochowa, Poland

**Keywords:** Image processing, Computer vision, Segmentation

## Abstract

Background segmentation is a process in which an algorithm removes the static background from an image. This allows only a changing section of the image. This process is important for motion detection or object tracking. In this article, an approach is proposed to compare several existing algorithms for background segmentation under severe weather conditions. Three weather conditions were tested: falling snow, rain and a sunny windy day. The test algorithms were executed on a test video containing frames collected by a dedicated Raspberry Pi camera. The frames used in the tests included cars, bicycles, motorcycles, people, and trees. Preliminary results from these tests show interesting differences in detail detection and detection noise.

## Introduction

**Figure 1:**
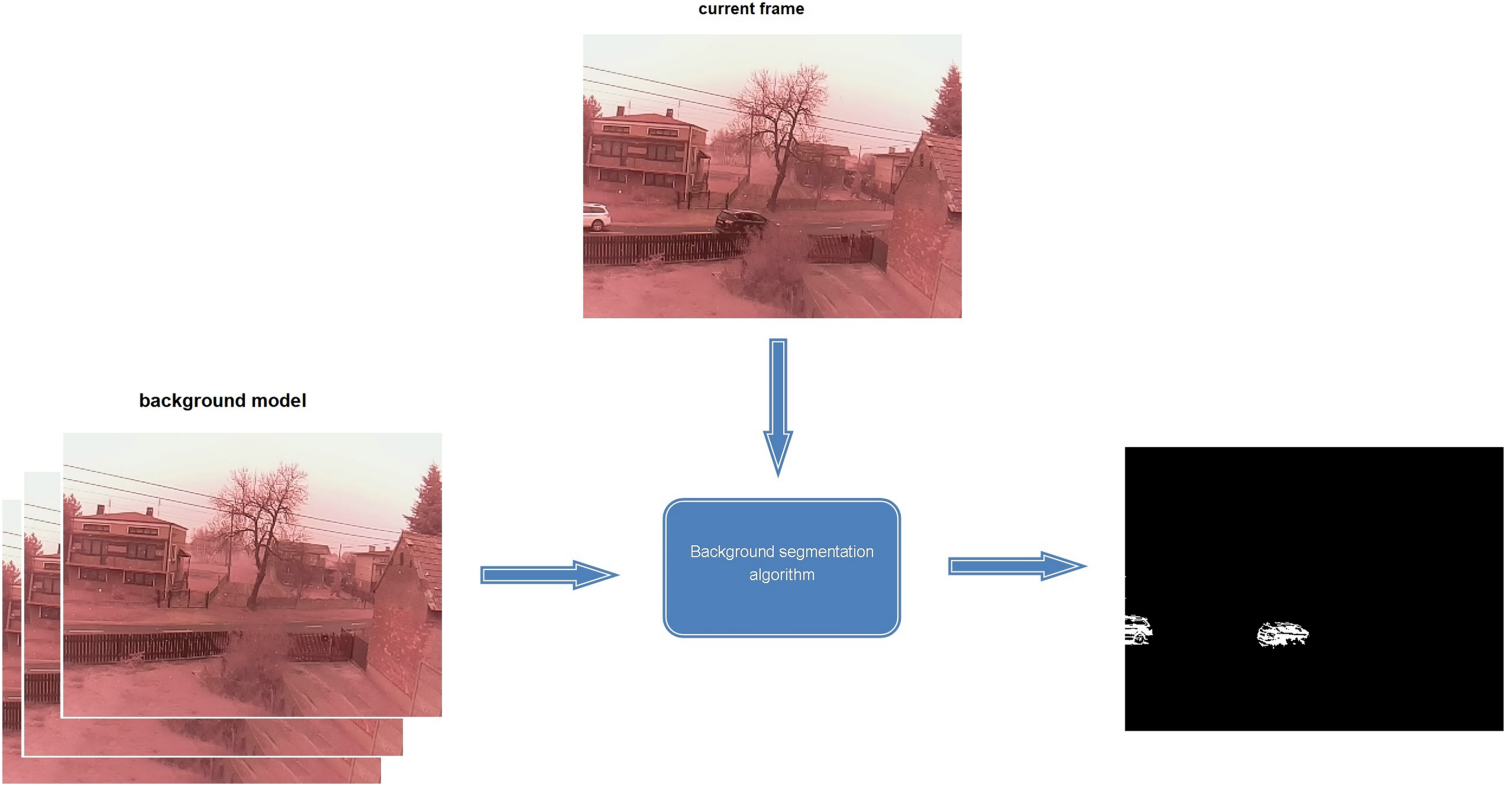
Overview about algorithm of background segmentation.

Background segmentation is a process in which an algorithm removes the static background from an image (see Fig. 1). This allows only a changing section of the image. This process is widely used in monitoring systems because it can easily detect a new object and track its movement. In addition, the development of mobile terminals and the Internet has led to an exponential growth in video data. To effectively analyse and use video, it is essential to analyse its content. For this, automatic methods of image segmentation into background and objects are necessary.

In this article, a comparison of the performance of the algorithms during severe weather conditions is proposed. This will evaluate how much noise can be obtained only by harsh weather. The severe weather conditions considered in this article were snow, rain and a windy day. The footage used to test the algorithms included moving vehicles such as cars, motorcycles and bicycles, moving pedestrians, trees and power lines waving in the wind. The results of these tests will allow the determination of the algorithm that was most resistant to weather-related disturbances and the algorithm that was most susceptible to disturbances and was the most accurate in analysing the changing image. All algorithms were used with default parameters (LearnOpenCv, 2021a, 2021c).

In the article, we propose a modification of the background segmentation algorithm. In the first step, our method uses the operations known from the GMG algorithm. However, the second step proposes a change in the form of a change in the method of pixel filtration and a change in the method of eliminating interference. The proposed changes result in the improvement of segmentation efficiency. A new algorithm of background segmentation is our contribution, and we compare our algorithm with other popular, and results of experiments are presented in “Introduction”.

## State of the Art

### Background segmentation algorithms

The first methods of image segmentation mainly used geometrical features of objects. The classic background removal method analyses all pixels in the image and treats those quickly changing as objects in the foreground. Any significant change in the image and background model represents a moving object. Pixels belonging to an area that have changed are candidates for further analysis (Jain & Grauman, 2014). The algorithm of connected components is used to estimate the areas belonging to objects. The extraction of objects occurs based on comparing the image with the scene model and then finding deviations from the model for each input frame (Stauffer, Eric & Grimson, 2000; Yao et al., 2020). Background subtraction was proposed by Wren et al. (1997). They used a multiclass statistical model of a pixel in a stationary background. For each pixel in the input video frame, the probability that the pixel was in the foreground was computed. In Elgammal et al. (2002), Elgammal and Davis proposed the use of Kernel Density Estimation (KDE) to represent background pixels. They created a background statistical model that detected moving objects in the scene. Han & Davis (2012) proposed background modelling and a technique for its removal using a multiple feature function, where the generative (Kernel Density Approximation (KDA)) and discriminant (Support Vector Machine (SVM)) techniques were used for classification. Irani, Rousso & Peleg (1994), to detect and remove the background, detected and tracked blocking and transparent moving objects and then applied temporal integration that keeps the tracked object in focus while blurring the rest of the image. Criminisi et al. (2006) presented the segmentation of movies using the *a priori* probability of combining motion, colour and contrast with spatial and temporal arguments. They created a mechanism for automatic layer separation and background replacement. Barnich & Van Droogenbroeck (2011) introduced a background subtraction algorithm based on samples. This method creates a pixel model and classification process and initializes and updates it in the background.

### Algorithms used in experiment

The first algorithm used to extract the background from an image was CouNT (CNT) (Github, 2021). Its performance is intuitive because it is comparable human perception of background. If something in the field of view does not change for a certain amount of time, the human brain considers it as part of the background. CNT counts the number of frames per second for which a pixel value remains unchanged (Zeevi, 2021). A special threshold was applied to all pixel value changes to eliminate false detections for changing illumination. To determine the threshold, a number of tests were performed on cloudy days when the lighting conditions change dynamically. The algorithm code was been optimized and, according to the developer’s tests, runs about 2.5 times faster Code (2021) on low-end ARM processor hardware such as the Raspberry Pi 3 than the MOG2 algorithm. For powerful processors the performance difference was almost invisible. The algorithm can run in parallel and the default value of parameter was parallel. The first value minPixelStability describes the number of frames used by the algorithm to create the background model. The recommended value for this parameter is the number of frames per second (FPS) in the recording/stream. The algorithm requires the maxPixelStability parameter to be specified which is responsible for the background update time. It is the number of frames needed for the pixel value to be recognized as the new background. The author’s tests have shown that the best results are obtained by setting it to 60 s, *i.e.*, the number of FPS*60. The last parameter was History, set to false, allows to ignore the value of maxPixelStability. This option may be needed when analysing very fast changing scenes. However, it may generate noise.

Mixture of Gaussian’s (MOG) (KaewTraKulPong & Bowden, 2002) was the second algorithm studied. It is a method in which each background pixel value *x*_*N*_ is modelled by a mixture of k Gaussian distributions at time *N*:

(1) $$p({x_N}) = \sum\limits_{j = 1}^K {\omega _j}\eta ({X_N};{\theta _j})$$

Here: *ω*_*j*_ is the weight parameter of the *k*^*th*^ Gaussian component. *η*(*X*;*θ*_*k*_) is the normal distribution of *k*^*th*^ component. Different distributions represent each of the background and foreground colours. The weight of each of these distributions in the model was proportional to the dwell time of each colour on a given pixel. Therefore, when the weight of a distribution for a given pixel is low, that pixel is classified as foreground. In the OpenCV library, the algorithm takes the following default values.

History means the number of frames that will be used to accumulate weights on the model, throughout the processing period. A short history results in increased sensitivity to sudden changes in illumination. The second parameter, nmixtures, specifies the number of Gaussian distributions to be used. The third parameter, backgroundRatio, determines the threshold weight based on which we distinguish the background from the foreground. A low threshold will create noise or false objects. The last parameter is noiseSigma which is responsible for the noise level. High values prevent noise or false objects.

The next MOG2 (Zivkovic, 2004) algorithm is an improved version of the MOG algorithm. The main limitation of the MOG algorithm, the fixed number of distributions, was improved. The MOG2 used a variable number of Gaussian distributions that were mapped pixel by pixel. The algorithm has only three configurable parameters.

The first parameter history determines the length of the history in the algorithm. The second varThreshold is the threshold of the square of the Mahalanobis distance between the pixel and the model. This describes whether the pixel is correctly described by the background model. The last parameter which is detectShadows is the shadow detection flag. If it is set to false, the algorithm runs a bit faster.

The K-nearest neighbors (KNN) (Zivkovic & van der Heijden, 2006) algorithm was also investigated. The algorithm used the K-nearest neighbour method for background subtraction. The method is very efficient in cases where the number of foreground pixels is small. The algorithm through recursive equations, performs a continuous update of the parameters of the Gaussian mixture model and to simultaneously select the correct number of components for each pixel. The method has three configurable parameters. The first one is history, which is the number of remembered states. The next one named dist2Threshold is threshold on the squared distance between the pixel and the sample to decide whether a pixel is close to that sample. The last one specifies whether the algorithm should detect shadows or not.

Another algorithm studied was Local SVD Binary Pattern (LSBP) (Guo, Xu & Qiang, 2016), which is an extension of LBP (Heikkila & Pietikainen, 2006) with a local singular value decomposition (SVD) operator. In the algorithm, each pixel was modelled and described separately using complex features. This description consisted of a new LSBP feature descriptor and colour intensity. The new descriptor performs very well in regions with dynamic lighting, various types of noise and shadows. In the method, each pixel *P*(*x*, *y*) is modelled by an array of N last observed background samples. The background model is described as *B*(*x*, *y*). The samples must contain *BInt*_*index*_(*x*, *y*) and *BLSBP*_*index*_(*x*, *y*).

(2) $$B(x,y) = {B_1}(x,y),...,{B_{index}}(x,y),...,{B_N}(x,y)$$

The index represents the sequence number of the background samples. *N* is the number of samples in the background model. *N* is used to balance the precision and sensitivity of the sample-based methods. In order to classify a pixel at the (x, y) coordinate, the current frame should be matched with their samples. To work properly, the algorithm needs to update the background changes. This is accomplished by the *T*(*x*, *y*) and is an update for each individual pixel. Only those pixels that were previously classified as background are updated. LSBP has a long list of configurable parameters. The first argument acts as a flag for camera motion compensation. The available values are stored in an enum.

The second argument, nSamples, specifies the number of stored samples for each point. The next, LSBPRadius, is the radius of the LSBP decryptor. The values Tlower, Tupper, Tinc, Tdec refer to the boundary and step for T-values. The next two values Rscale and Rincdec, determine the scaling of the threshold values and the increase or decrease of the step for the threshold values. noiseRemovalThresholdFacBG is for removing noise for points specified as background and noiseRemovalThresholdFacFG is for removing foreground noise. The threshold for a binary string can be specified using LSBPthreshold. The last parameter that defines the minimum number of matches for a sample to be foreground-qualified is minCount.

The Google Summer of Code (GSOC) algorithm (LearnOpenCv, 2021b) is an improved version of the LSBP algorithm. The new algorithm is faster and more resistant to noise. The main change is to drop the LSBP descriptors in favor of RGB color values. Another change is the detection of pixels that often move from background to foreground and vice versa. In the final stage, the algorithm removes noise and applies a Gaussian blur filter. The parameters of this algorithm are similar to LSBP.

The first and second parameters are exactly the same as for LSBP algorithm and specify, in sequence, the camera motion compensation flag and the number of retained frames for each point. The next replaceRate determines how quickly the model is updated. The neighbor propagation probability is the propagationRate parameter. The hitsThreshold parameter value is responsible for the number of positives before being considered replaceable. The scale factor and bias factor for the threshold are the alpha and beta parameters respectively. The next parameters are related to the aforementioned blinker. The first blinkingSupressionDecay is the distribution factor for blink suppression and the second blinkingSupressionMultiplier is the multiplier for blink suppression. The last parameters deal with the noise removal strength for background noiseRemovalThresholdFacBG, and for foreground noiseRemovalThresholdFaceFG.

The last algorithm we compare in the experiment is the Self-Organizing method (SOM) described in Maddalena & Petrosino (2008). The authors proposed an approach based on self-organization through artificial neural networks. The proposed approach could handle scenes that include moving backgrounds, gradual changes in lighting, and camouflage. The technique has no preprocessing limitations and is resistant to shadows cast by moving objects. The presented results confirm the high efficiency of background detection for various types of films.

## Methods

We proposed modifying the GMG algorithm (Godbehere, Matsukawa & Goldberg, 2012; Python, 2021). It is a combination of static background image estimation, Kalman filter bank, Gale-Shapley matching and Bayesian segmentation performed for each pixel. The implementation of the algorithm allows selective filtering of traces based on dynamic data.

The new proposed algorithm is an improvement of the GMG algorithm. As mentioned earlier, we can distinguish two stages of operation in the GMG algorithm. The first one, concerning the weighted value for each pixel, has not been modified. The second stage is filtering the pixels to reduce the amount of noise. In the original, only a median filter was used. As a result, harsh weather conditions leave a large amount of noise in the output image. In the proposed enhancement, besides the median filter, which is performed first, two morphological transforms are applied. The tests performed showed very good noise reduction results obtained from the first stage of the algorithm by using a combination of erosion and dilation. The first method after performing the median filter is erosion. The kernel is square-shaped and has the same size as the median filter. The erosion is based on a local minimum in the kernel area, this allows for a reduction in the detected areas which is equivalent to a reduction in the amount of noise. The last transformation in the filtering process is the opening filter. This filter involves the use of erosion followed by dilation. Dilation, unlike erosion, is based on a local maximum. Filtering using such methods allows the result to be cleaned of the noise that is generated in the first stage of the operation. Due to the last filter applied, the shapes of the objects are not significantly deformed by the preceding erosion filters (Bobulski, 2016; Bobulski & Kubanek, 2012).

The number of frames needed to initialize the correct operation of the algorithm is specified as initializationFrames. The second parameter decisionThreshold is the pixel transition threshold between classification as background or foreground. The higher the value, the higher the threshold for a pixel to become foreground more homogeneous.

## Research

### Algorithms during different weather conditions-own images

Our long-term research aims to develop a computer vision system for analysing the vehicle’s surroundings on the road. It is part of a far-reaching autonomous vehicle project. Therefore, the background segmentation and object detection algorithm should be effective in various weather conditions. The research scenario is a consequence of this assumption.

#### Snowfall

**Figure 2:**
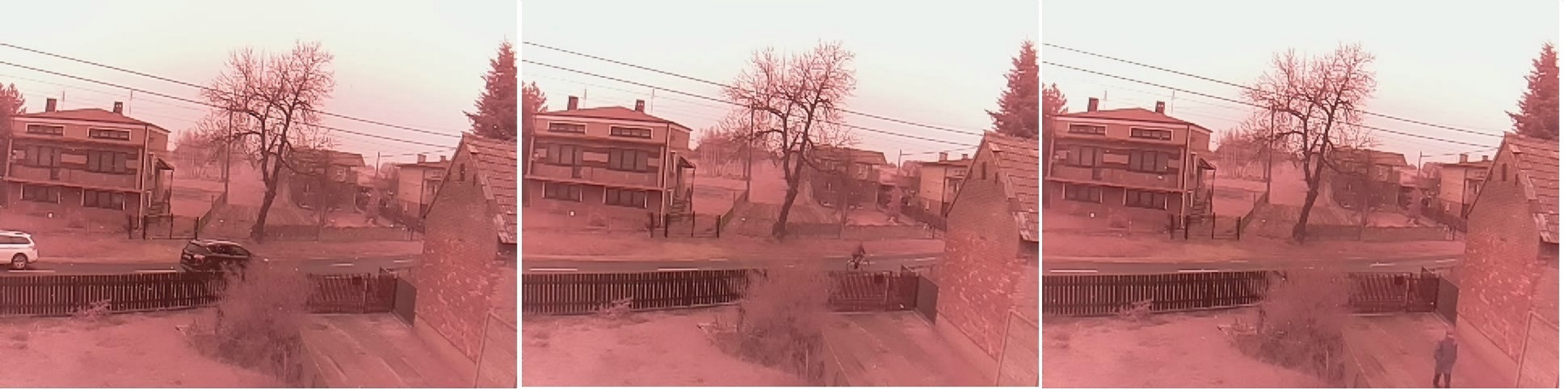
Original frames during snowfall.

The test snowfall frame (see Fig. 2) contains two vehicles, cyclist and a person. In the first image from the left, one, black, vehicle is in the center and the other, white, is on the left side of the image. The vehicle in the center is partially obscured by bushes. The image also includes trees and power lines that move in the wind. The middle image shows the cyclist on the right. The far right image contains the person in the bottom right corner

**Figure 3:**
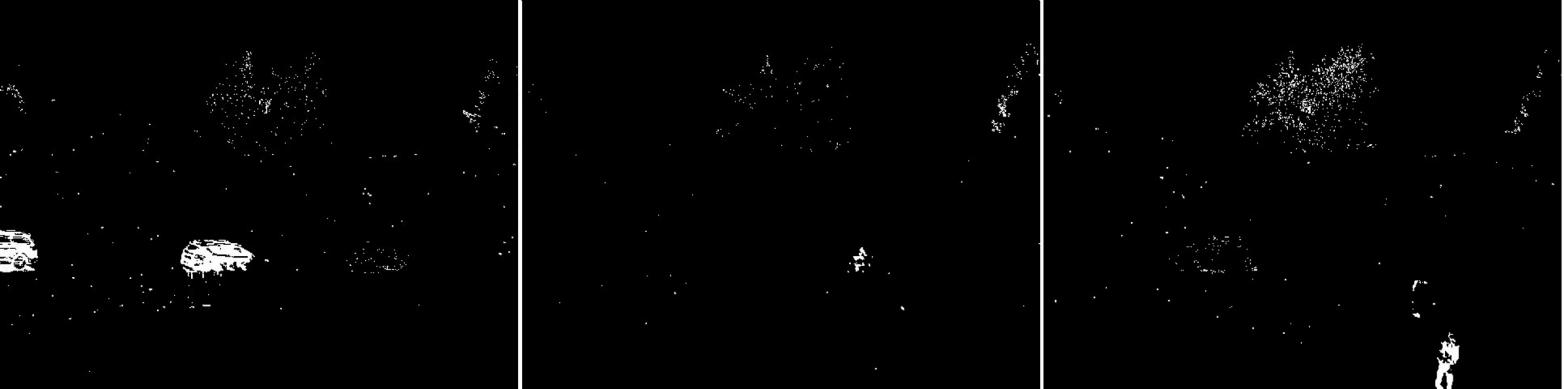
Results of CNT algorithm during snowfall.

The first algorithm for which results are presented is CNT (see Fig. 3). The vehicles are very well represented in this algorithm. Several elements such as wheels, windows, lights can be distinguished. Trees are shown as point clouds but they are not very accurately represented. Slight noise throughout the image is caused by snowflakes. In the case of the bicycle, the mapping is of very poor quality because the object cannot be recognized correctly. There is relatively little noise due to snow in this image. The outline of the trees can also be seen. In the last image, you can notice a fairly well rendered person. The tree in the middle is well represented.

**Figure 4:**
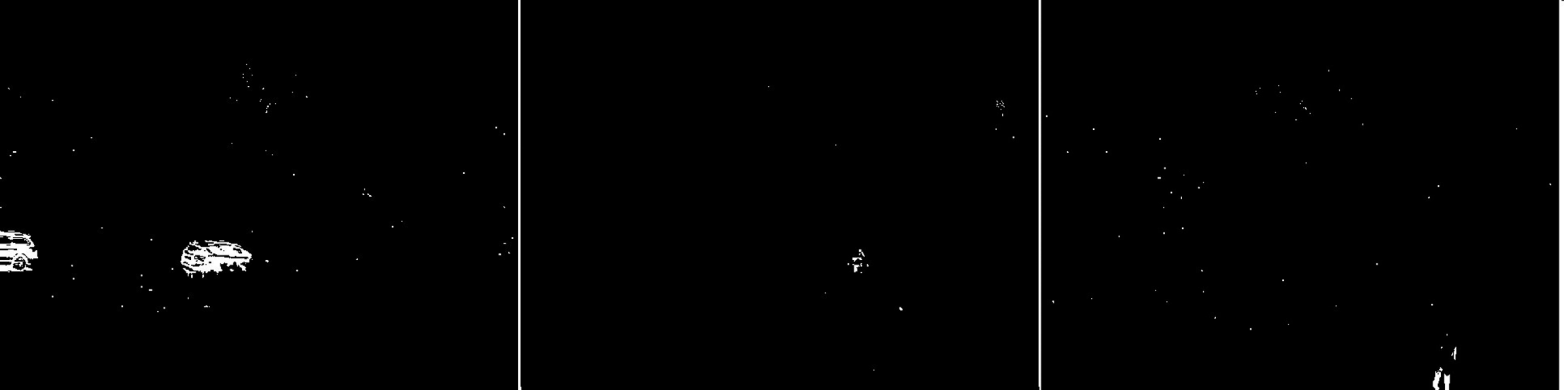
Results of MOG algorithm during snowfall.

In the second scenario, the MOG algorithm was used (see Fig. 4). On the first image, this algorithm also accurately represented both vehicles for which individual elements can be distinguished. The trees generate almost no noise. The amount of noise due to snowfall is small. The result for the middle image where the cyclist is located is of the same poor quality as the previous algorithm. The cyclist cannot be correctly recognized. Few points marking the trees. In the last image the pedestrian also cannot be recognized correctly. Noise reflecting trees and snow.

**Figure 5:**
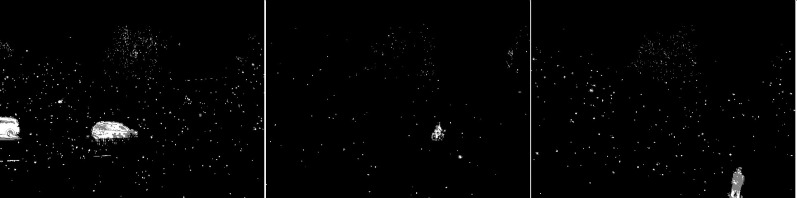
Results of MOG2 algorithm during snowfall.

The third algorithm analyzed is an improved version of MOG, the MOG2 (see Fig. 5). This algorithm is one of the most sensitive and accurate of the algorithms studied. The vehicles are accurately represented in the first image, along with the shade of gray that the shadows are labeled with. The tree located in the center is well represented. High noise due to snowfall is also visible. The middle image contains a clear representation of the cyclist. The two wheels of the bicycle can be recognized. The trees are also rendered. Noise due to snowfall also appears. The last image contains a fairly well rendered figure, but most of it is gray indicating a potential shadow. There is a lot of noise in the background due to snowflakes.

**Figure 6:**
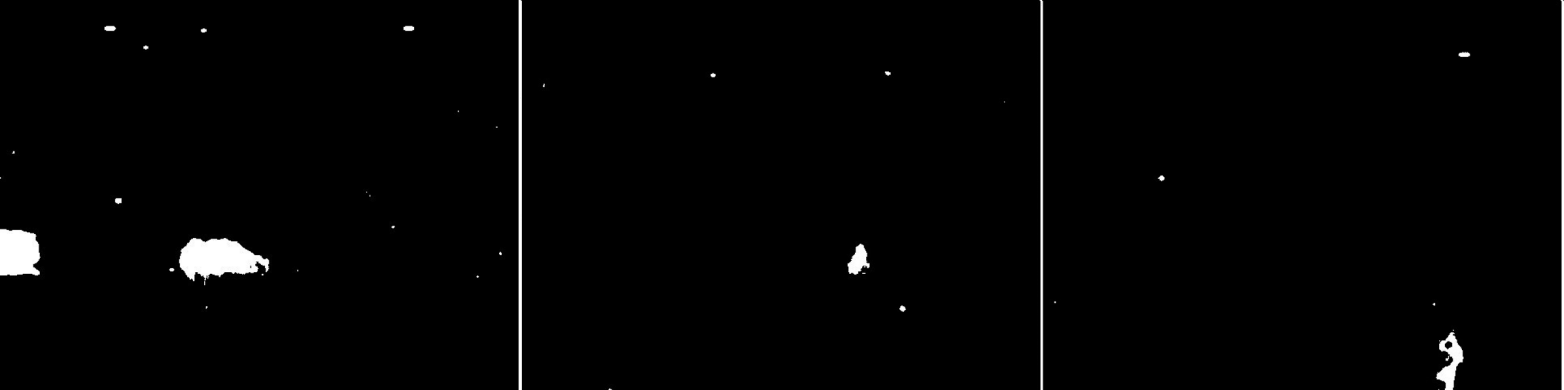
Results of GMG algorithm during snowfall.

The GMG algorithm was used in the fourth scenario (see Fig. 6). The result of the algorithm is an image containing several objects. The vehicles are large white blobs for which no individual elements can be identified as in previous algorithms. The cyclist is a fairly large white field in the image, but it cannot be dissected by the shape of the object. The image contains only a few noises besides the cyclist. For the last image, the pedestrian is also depicted as a blob that does not resemble him in shape. This image also contains only a few noises besides the main object. The algorithm seems like a good idea when is needed to detect large moving objects. The algorithm is not susceptible to noise.

**Figure 7:**
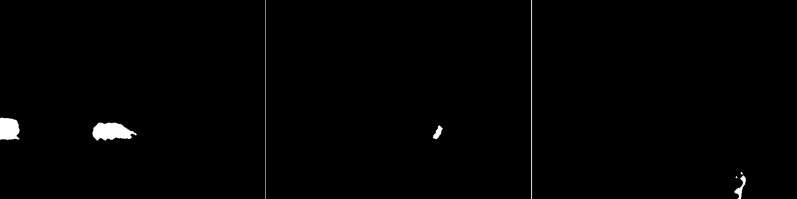
Results of updated GMG algorithm during snowfall.

An updated version of the GMG algorithm can be seen in Fig. 7. The results are better than for the original GMG. In all the resulting images, the noise is correctly cleaned. The filtering did not significantly affect the shape of the main objects.

**Figure 8:**
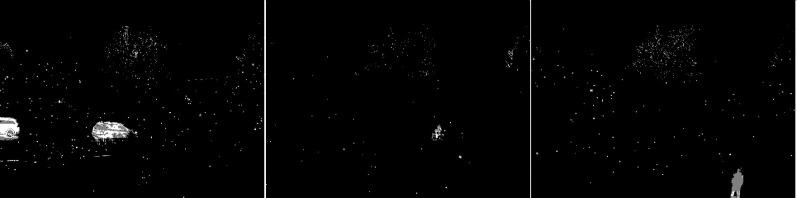
Results of KNN algorithm during snowfall.

The fifth algorithm KNN (see Fig. 8), is the second most accurate algorithm among those studied. The vehicles are very accurately mapped. Trees are also visible as clusters of points. Snowfall is visible in the output image as small groups of pixels although there are fewer of them than with the MOG2 algorithm. In the middle image, you can recognize the cyclist from the shape. It is not as well rendered as in MOG2, but this does not prevent you from classifying the object correctly. The trees are adequately visualized, and there is little additional noise. The last image shows a well rendered pedestrian, as with MOG2, in gray. There is much more noise due to precipitation in this image than in the middle image. The tree located in the middle is well rendered.

**Figure 9:**
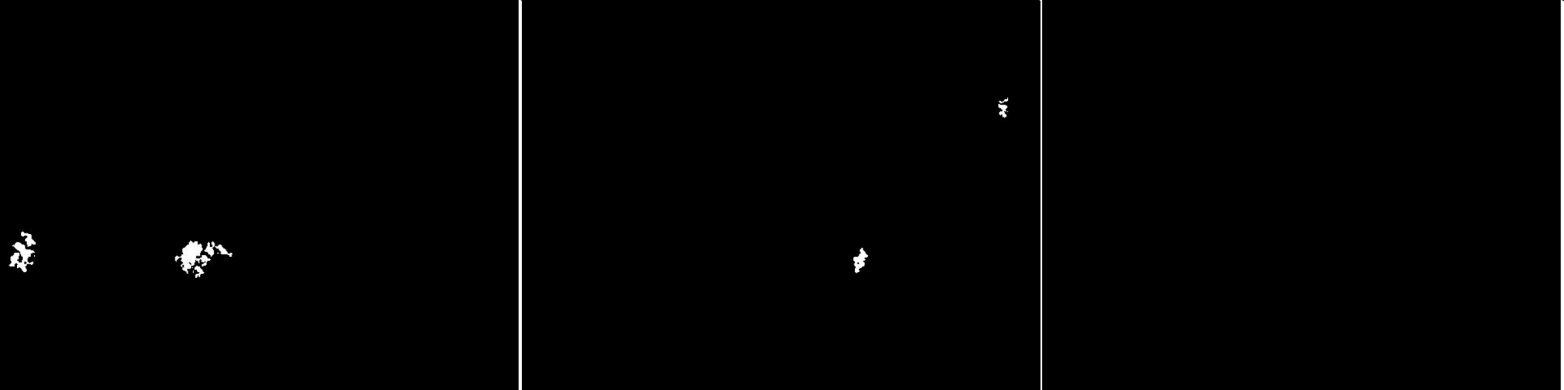
Results of LSBP algorithm during snowfall.

In the sixth scenario, the LSBP is presented in Fig. 9. The result was images without snowfall interference just like the GSOC algorithm. The difference between these algorithms can be seen in the detection of vehicles, bicycle and person. For this algorithm, the blobs are very jagged and their shape cannot be determined. They are smaller than the vehicles in the original image. This algorithm did a great job with the weather conditions, but the visualization of the detected objects are of very poor quality.

**Figure 10:**
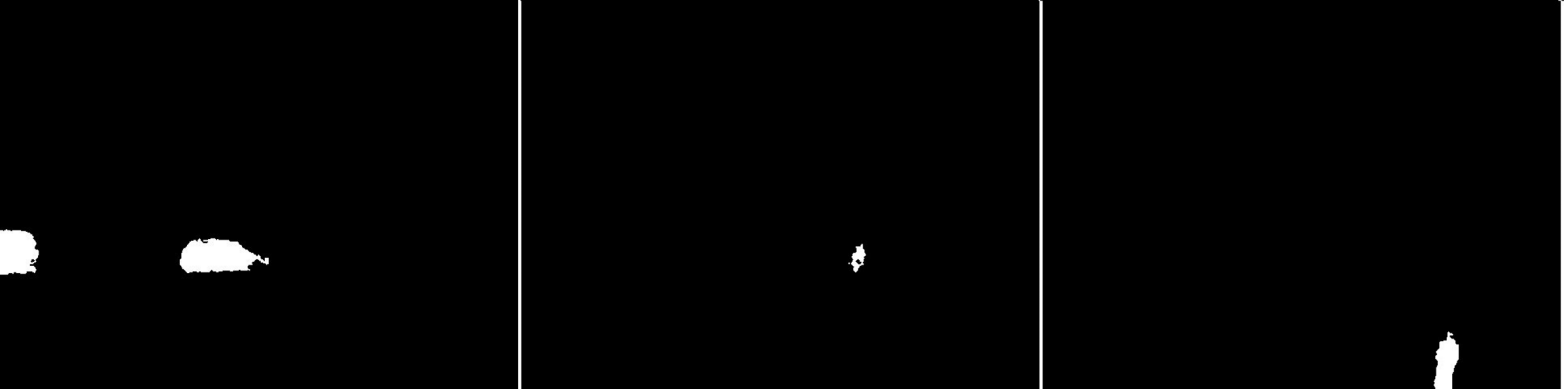
Results of GSOC algorithm during snowfall.

The seventh studied algorithm is GSOC (see Fig. 10). A noise-free image was obtained as the result. Only two blobs defining the position of the vehicles are visible. The cyclist and pedestrian are also the only recognized objects. Their shapes are not perfect, making it impossible to correctly classify these objects with only the result. No other objects are detected in the images, which means that it is the algorithm with the best resistance to weather-related interference among the tested algorithms.

**Figure 11:**
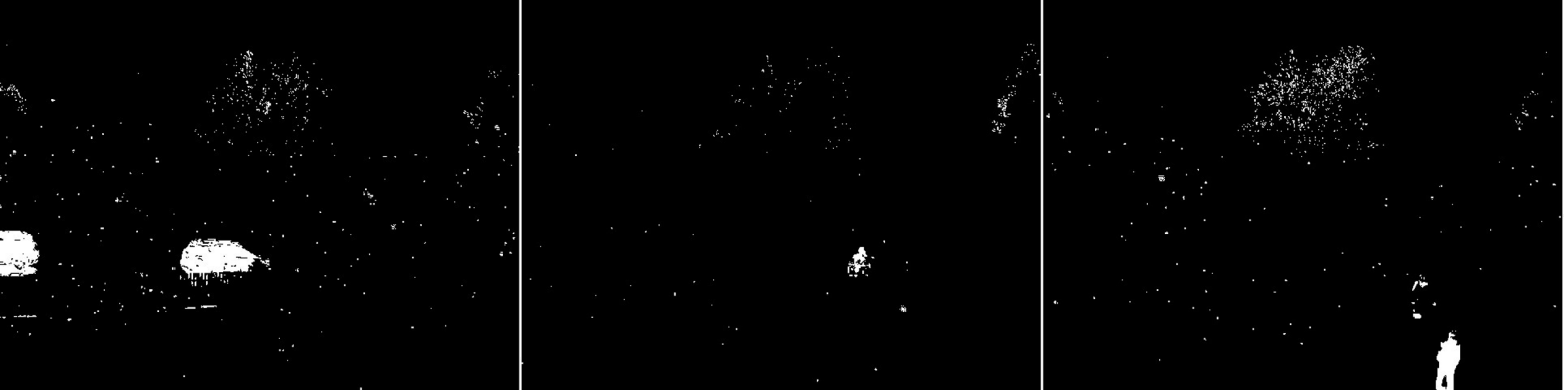
Results of SOM algorithm during snowfall.

The next used algorithm was SOM (see Fig. 11). The vehicles are very accurately mapped. Trees are also visible as clusters of points. Snowfall is visible in the output image as small groups of pixels although there are fewer of them than with the KNN and MOG2 algorithm. In the middle image, you can recognize the cyclist from the shape. It is not as well rendered as in MOG2 or update GMG. The trees are good visualized, and there is little additional noise. The last image shows a well rendered pedestrian, as with MOG2, in gray. There is much more noise due to precipitation in this image than in the middle image. The tree is well rendered.

#### Windy and cloudy

**Figure 12:**
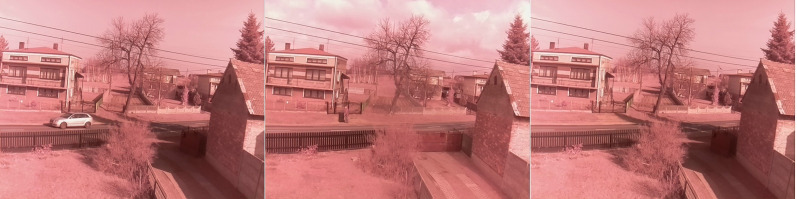
Original frame during windy and cloudy weather.

The next test of the algorithms was performed on recordings from a windy day (see Fig. 12). An additional complication is the high cloud cover, which is mainly visible in the middle image. The first image from the left shows a passing car. It is clear from the video that trees and power lines are moving. This should be picked up by the algorithms. The middle image, mentioned earlier, shows a person in addition to the cloud cover. The last picture shows two birds flying, they are on the right side of a tree. The small birds are a test of noise removal in the algorithms.

**Figure 13:**
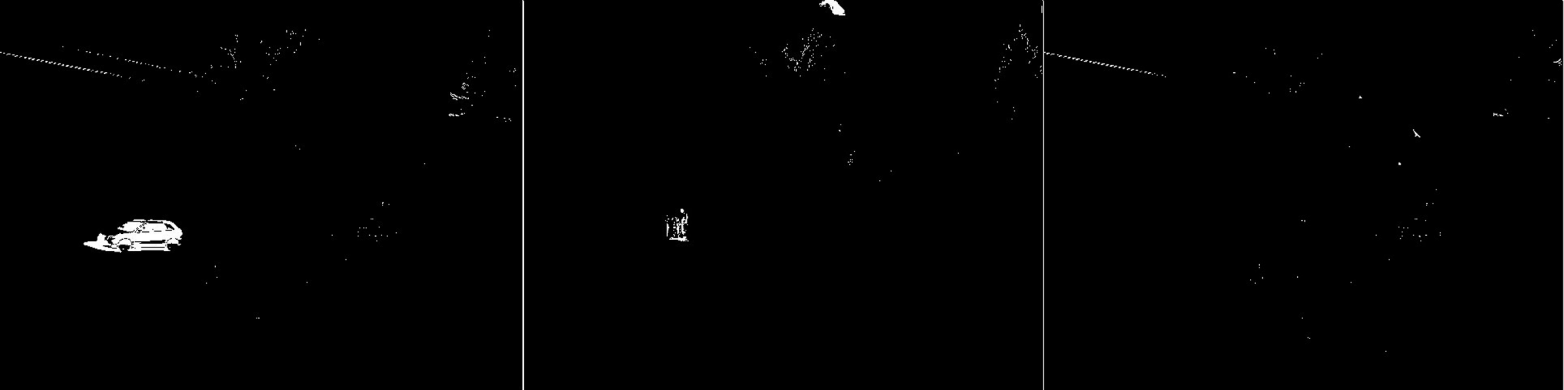
Results of the CNT algorithm during windy and cloudy weather.

For the first scenario under these conditions (see Fig. 13) and the first image from the left the CNT algorithm reproduced the car well. In addition, two power lines can be seen, which as mentioned were moving in the wind. However, the trees are not very visible. From the second resulting image, it is hard to deduce that the detected object is a person. This is due to the poor representation of shapes. Trees are more visible than in the previous image. The noise in the middle of the top edge of the image is also visible. It is caused by cloud cover. The last image shows almost zero representation of the trees. A single power line is visible. There are also birds flying in the picture.

**Figure 14:**
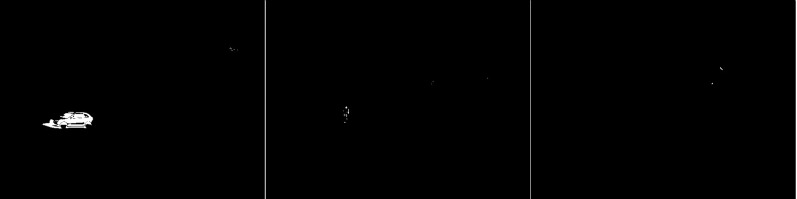
Results of the MOG algorithm during windy and cloudy weather.

The second scenario is to analyse the results of the MOG algorithm (see Fig. 14). The result presented in the first image is just a well-mapped vehicle. Only a slight perturbation near a tree is visible, but trees were not detected. Power lines were not recognized. The middle image showed only a poorly rendered person. There is no interference from cloud cover. The third image shows very good results of the algorithm. Only flying birds are visible. No additional interference’s.

**Figure 15:**
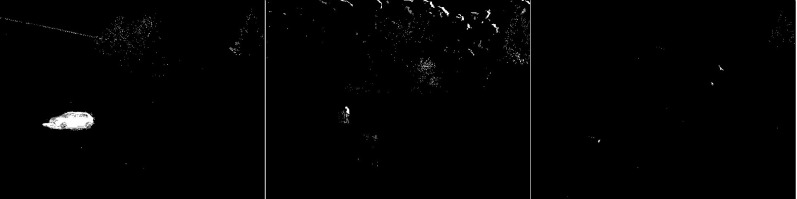
Results of the MOG2 algorithm during windy and cloudy weather.

The third scenario shows the detection accuracy for the MOG2 algorithm (see Fig. 15). The first image shows a power line, unfortunately the second one was not detected. The trees are well mapped. The vehicle is also correctly reconstructed. Additionally, there is some noise at the bottom of the image. The middle image is a well rendered figure standing sideways. The trees are also visible. However, due to the high detection accuracy, a significant amount of noise was created. The noise is reproduced by moving clouds. The last image contains not only well rendered birds, but additionally a tree on the right. Moreover, there is some noise at various locations.

**Figure 16:**
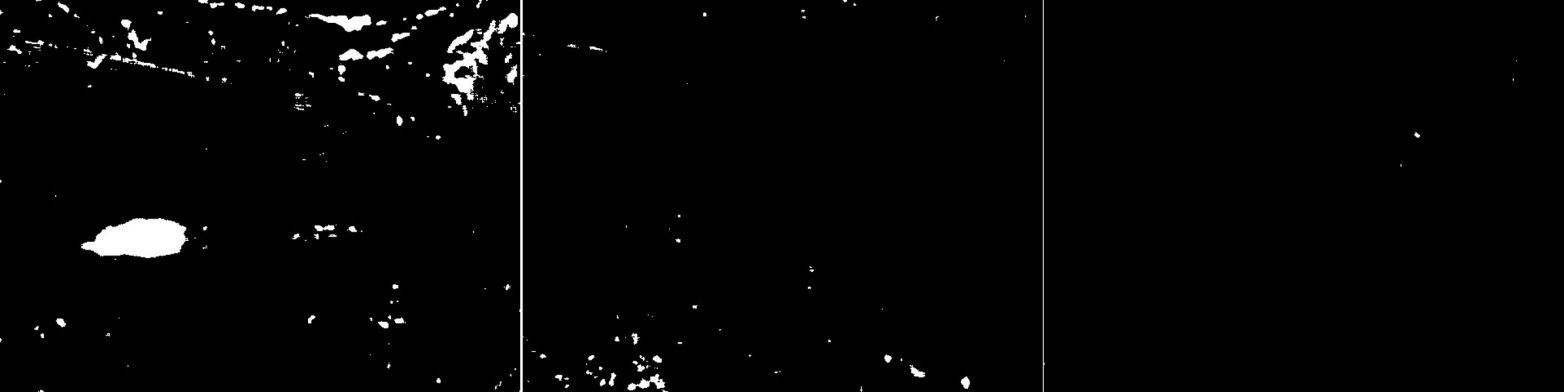
Results of the GMG algorithm during windy and cloudy weather.

The outputs for the fourth scenario are the results of the GMG method (see Fig. 16). The first image shows a vehicle, power lines, and a tree. However, the result is very poorly readable due to the large amount of additional noise located almost everywhere. The second image does not contain any characters, but only the noise at the top and bottom of the image. The last result of the method showed the birds correctly.

**Figure 17:**
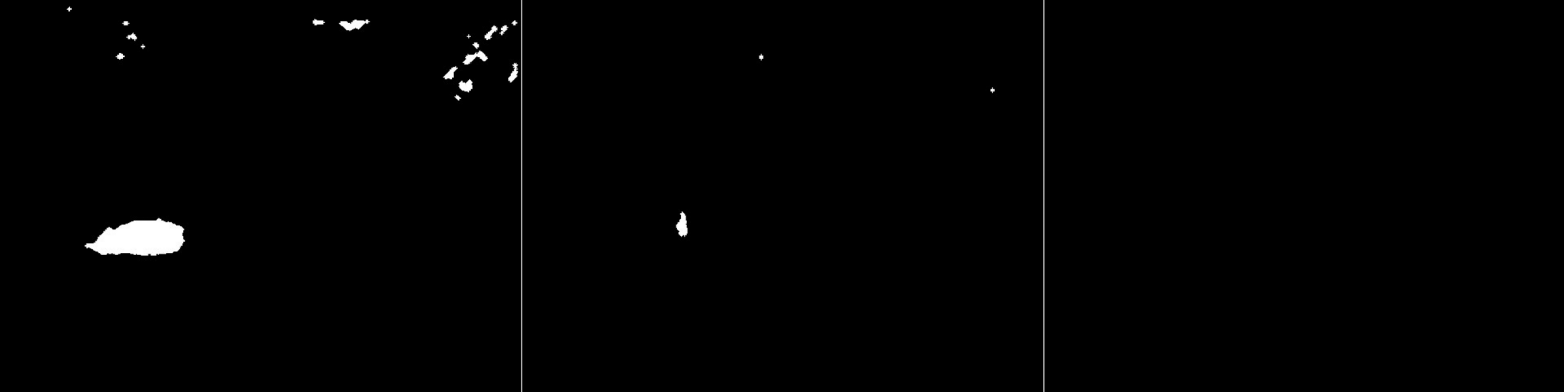
Results of updated GMG algorithm during windy and cloudy weather.

An updated version of the GMG algorithm is shown in Fig. 17. The results are again better than for the original GMG. The image with the vehicle has only a small amount of noise at the top of the image. The vehicle has a very similar shape to the original, but the moving power lines have been lost. In the second image, a significant improvement is achieved as a person is detected that was not present in the original. For the third image, unfortunately a worse result was achieved because small birds were removed by filtering.

**Figure 18:**
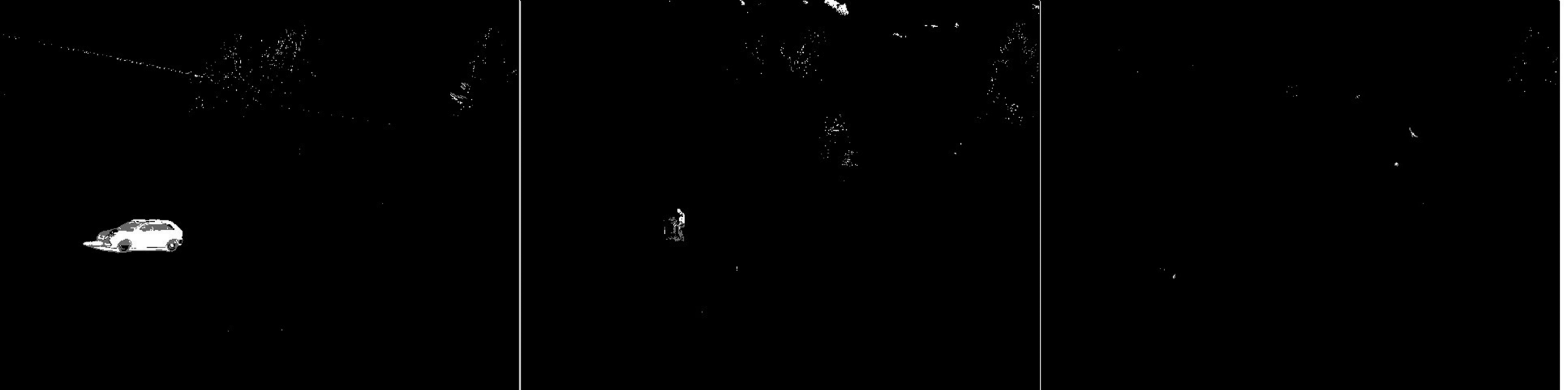
Results of the KNN algorithm during windy and cloudy weather.

In the fifth scenario, the good performance of the KNN method can be admired (see Fig. 18). The recovery of the vehicle located in the first image is very good. The trees are also correctly represented. With the power lines it is exactly like the MOG2 algorithm, one is missing. The second image is a correctly depicted figure and trees. There is definitely less cloud cover interference in this result than in MOG2. The results of the last one are also very similar to the compared method. The birds can be seen correctly, plus the tree. There are also few disturbances.

**Figure 19:**
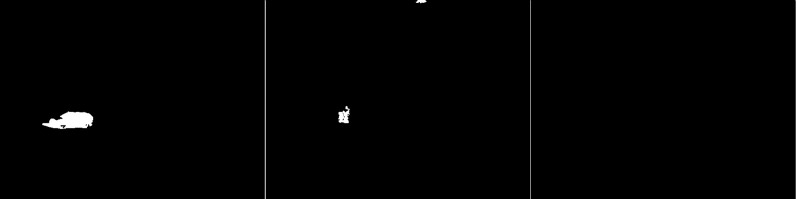
Results of the LSBP algorithm during windy and cloudy weather.

The sixth case is the LSBP method and as you can see from the results (see Fig. 19). This method is very resistant to interference. The first image shows only the vehicle. Moving trees or power lines were considered part of the background. The second image shows a spot located in place of a person. It is impossible to tell from the shape what is presented in the resulting image. There is also a distortion at the top of the image. The third image contains nothing. The birds. probably because of their small size, were classified as a background.

**Figure 20:**
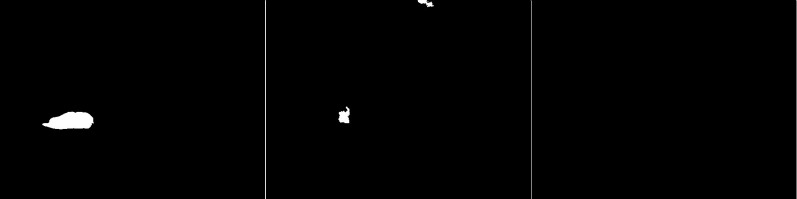
Results of the GSOC algorithm during windy and cloudy weather.

In the seventh scenario, the results of the GSOC are presented (see Fig. 20). It can be seen that also this algorithm is robust to interference. The first image shows only the vehicle. Everything else was considered as background. The next image is a blob to represent a person. As with the LSBP algorithm, the top of the image shows the interference from the cloud cover. Unfortunately, nothing was visualized in the last image. The birds also turned out to be too small for this algorithm.

**Figure 21:**
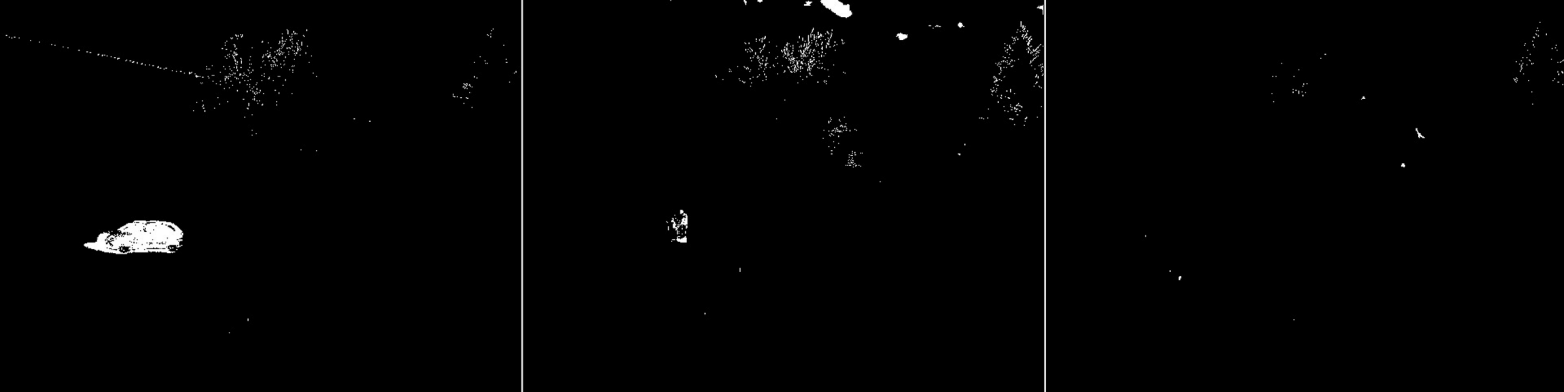
Results of the SOM algorithm during windy and cloudy weather.

In the last case, the results of the SOM are presented in Fig. 21. This algorithm is robust to interference. The first image shows the vehicle and power line. Everything else was considered as background. The next image is a patch to represent a person. The top of the image contains the interference from the cloud cover. The trees are well mapped. The middle image is a well rendered figure standing sideways. The trees are also visible. Due to the high detection accuracy, a significant amount of noise was created. The last image contains not only well rendered birds, but additionally a tree on the right.

#### Rainfall

**Figure 22:**
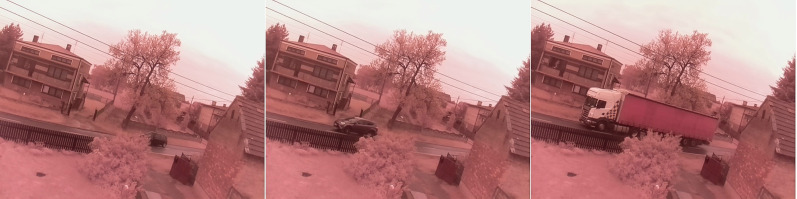
Original frame during rainy weather.

The last weather condition used to test the background clipping methods is rainfall. During this rainfall, the wind was not noticeable. For these conditions, frames containing cars were selected (see Fig. 22). One of them is a truck. It is an additional challenge, besides the falling rain, for the algorithms because it creates a large illumination change near itself. The first image shows only part of the vehicle. The second part is hidden in the bushes. In the second image, the vehicle is in the centre. The third image is the previously mentioned truck. The area around the centre of the vehicle is obscured by bushes.

**Figure 23:**
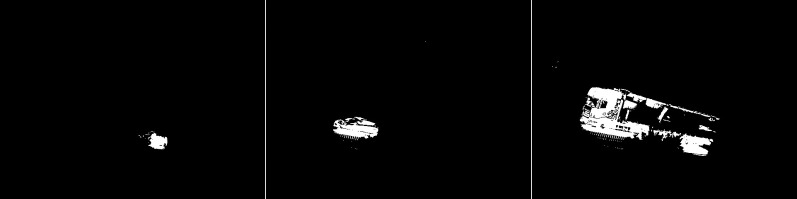
Results of the CNT algorithm during rainy weather.

The first case is the results of the CNT algorithm (see Fig. 23). The two initial images correctly represent the vehicles. No background noise is visible. In the last image, part of the truck trailer is omitted from detection. Other than that, the truck itself is well rendered with a lot of details. There is only some minor noise due to weather conditions.

**Figure 24:**
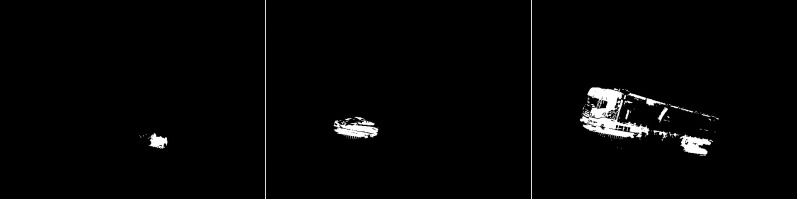
Results of the MOG algorithm during rainy weather.

The second case is the MOG method (see Fig. 24). In no image showing the result of the operation, no interference due to weather conditions can be seen. The first and second results images are almost identical to the previous algorithm. The vehicles are correctly identified. In the third image several details of the difference between the results for CNT can be seen. In this case the cab of the car is detected correctly, while for the semitrailer only an incomplete contour.

**Figure 25:**
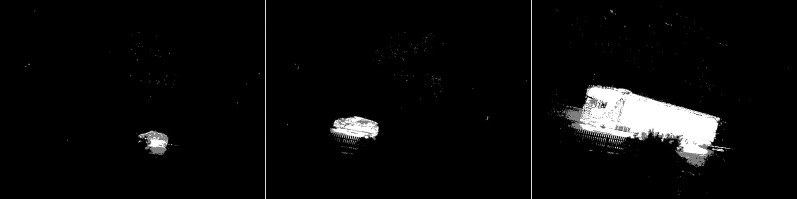
Results of the MOG2 algorithm during rainy weather.

Another case is the MOG2 algorithm (see Fig. 25). The first image shows a correctly detected part of the vehicle. The middle and right photos show the noise that can be detected rain. The second image shows the vehicle as well as part of the road underneath the car. There is noise in the center of the image, and there is little noise to the right and left of the image. In the case of the last photo, the algorithm correctly detected both parts of the truck and a fragment of the fence and bushes.

**Figure 26:**
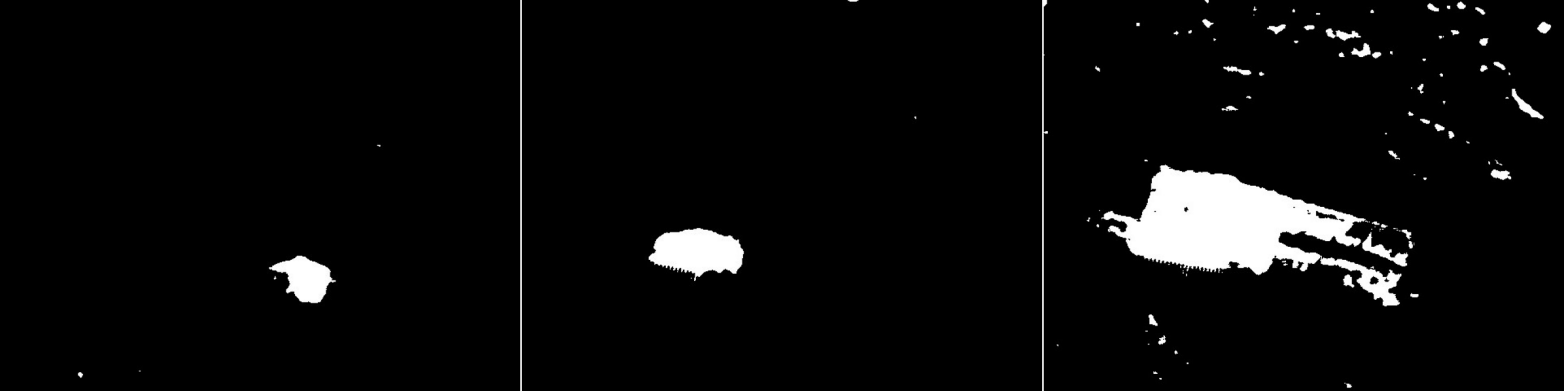
Results of the GMG algorithm during rainy weather.

The fourth results are the effects of the GMG method (see Fig. 26). In the first image we see a large blob that represents a moving car. In addition, we can see a couple of small blobs of noise. The middle image is another correct determination of the vehicle, without much noise in the image. The last image shows the poor result of the algorithm. The trailer of the vehicle is not detected correctly, Additionally there is noise in the upper part of the image. It may be caused by cloud cover. There is also some noise in the lower left corner, but it is impossible to determine its possible source.

**Figure 27:**
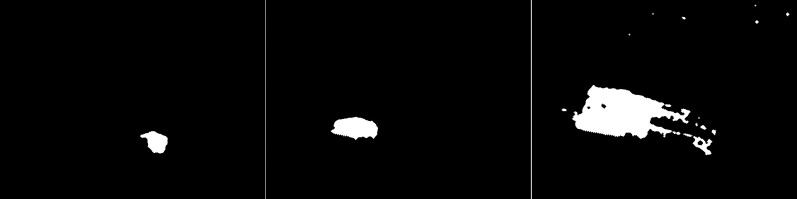
Results of updated GMG algorithm during rainy weather.

An updated version of the GMG algorithm is shown in Fig. 27. For the first image, the slight noise that was visible in the original is not present. The shape of the vehicle has changed slightly. The second photo is almost identical. For the last photo, the amount of noise present at the top has been significantly reduced. The truck trailer is slightly less well presented than the original.

**Figure 28:**
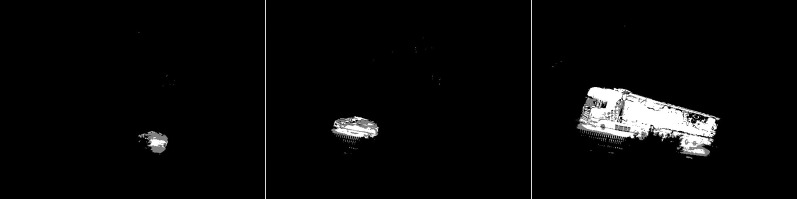
Results of the KNN algorithm during rainy weather.

The fifth case is the KNN algorithm (see Fig. 28). In all three images, there are fewer slight interference’s than the MOG2 method. The first image is a representation of a vehicle, and some noise in the middle that may have come from a tree. The second image also shows subtle noise at the tree location. The car, along with the fence section, was reproduced well. For the last image, the result of the activity contains both parts of the truck correctly shown. The image is very detailed. One can only notice a slight noise on the left side of the image

**Figure 29:**
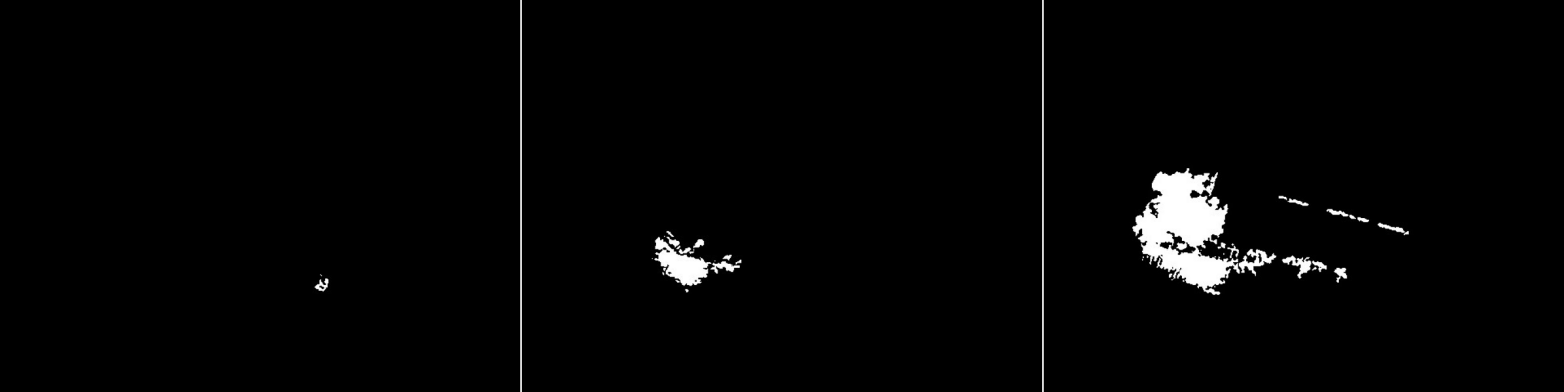
Results of the LSBP algorithm during rainy weather.

The sixth algorithm is LSBP (see Fig. 29). There is no interference in the output images. The first two images are poor detection of moving objects. The outlines are very jagged and definitely smaller than the actual moving foreground objects. In the last image, the car trailer has been severely ignored. Only a portion of the top and bottom edges are visible.

**Figure 30:**
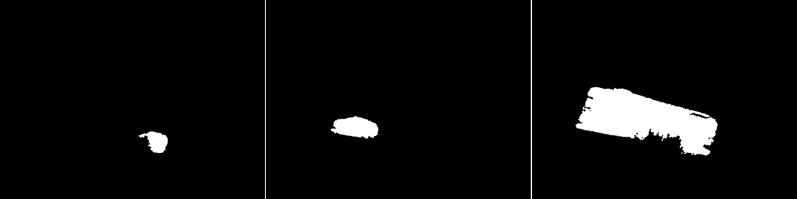
Results of the GSOC algorithm during rainy weather.

The next method is GSOC (see Fig. 30). As with the native LSBP algorithm, interference does not occur on the running results. In all cases, the foreground objects lack detail, but their size and shape are correct. Even for the last one, the third one, the vehicle and trailer are correctly detected.

**Figure 31:**
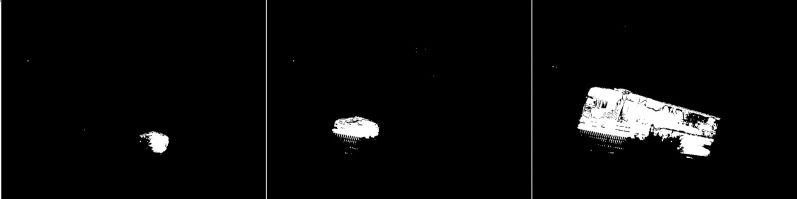
Results of the SOM algorithm during rainy weather.

The last method is SOM (see Fig. 31). Noises and interference does not occur on the running results. The foreground objects are good, and their size and shape are correct.

### Algorithms during different weather conditions-images from public database

#### Datasets

We use two public databases for ChangeDetection: Net CDNET (Wang et al., 2014) and BMC-Background Models Challenge (Vacavant et al., 2013). The first one consists of two datasets 2012 DATASET and 2014 DATASET. Both provide a realistic, camera-captured (no CGI), diverse set of videos. They have been selected to cover a wide range of detection challenges and are representative of typical indoor and outdoor visual data captured today in surveillance, smart environment, and video database scenarios. The 2012 DATASET includes the following challenges: dynamic background, camera jitter, intermittent object motion, shadows and thermal signatures. The 2014 DATASET includes all the 2012 videos plus additional ones with the following difficulties: challenging weather, low frame-rate, acquisition at night, PTZ capture and air turbulence. Each dataset is accompanied by accurate ground-truth segmentation and annotation of change/motion areas for each video frame.

In the second, they have proposed a complete benchmark composed of both synthetic and real videos. They are divided into two distinct sets of sequences: learning and evaluation. The benchmark is first composed of 20 urban video sequences rendered with the simulator. This dataset focuses on outdoor situations with weather variations such as wind, sun or rain. They also proposed some evaluation criteria and an associated free software to compute them from several challenging testing videos.

#### Snowfall

The snow weather condition images were used to test first. For these conditions, frames containing cars and people were selected (see Fig. 32). The results of experiments on images during snowy weather are presented on Figs. 33–41.

**Figure 32:**
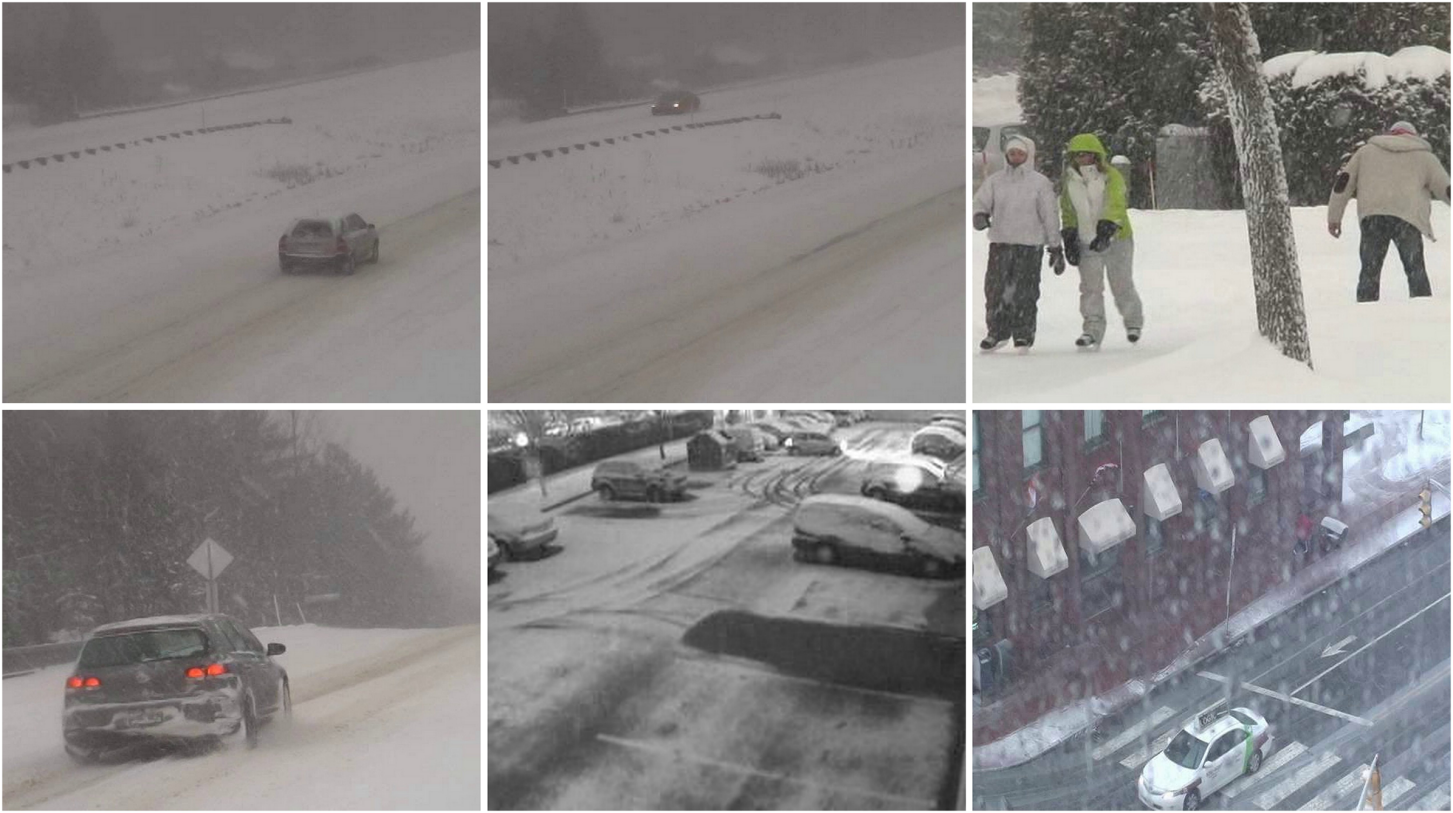
Original frame during snow.

**Figure 33:**
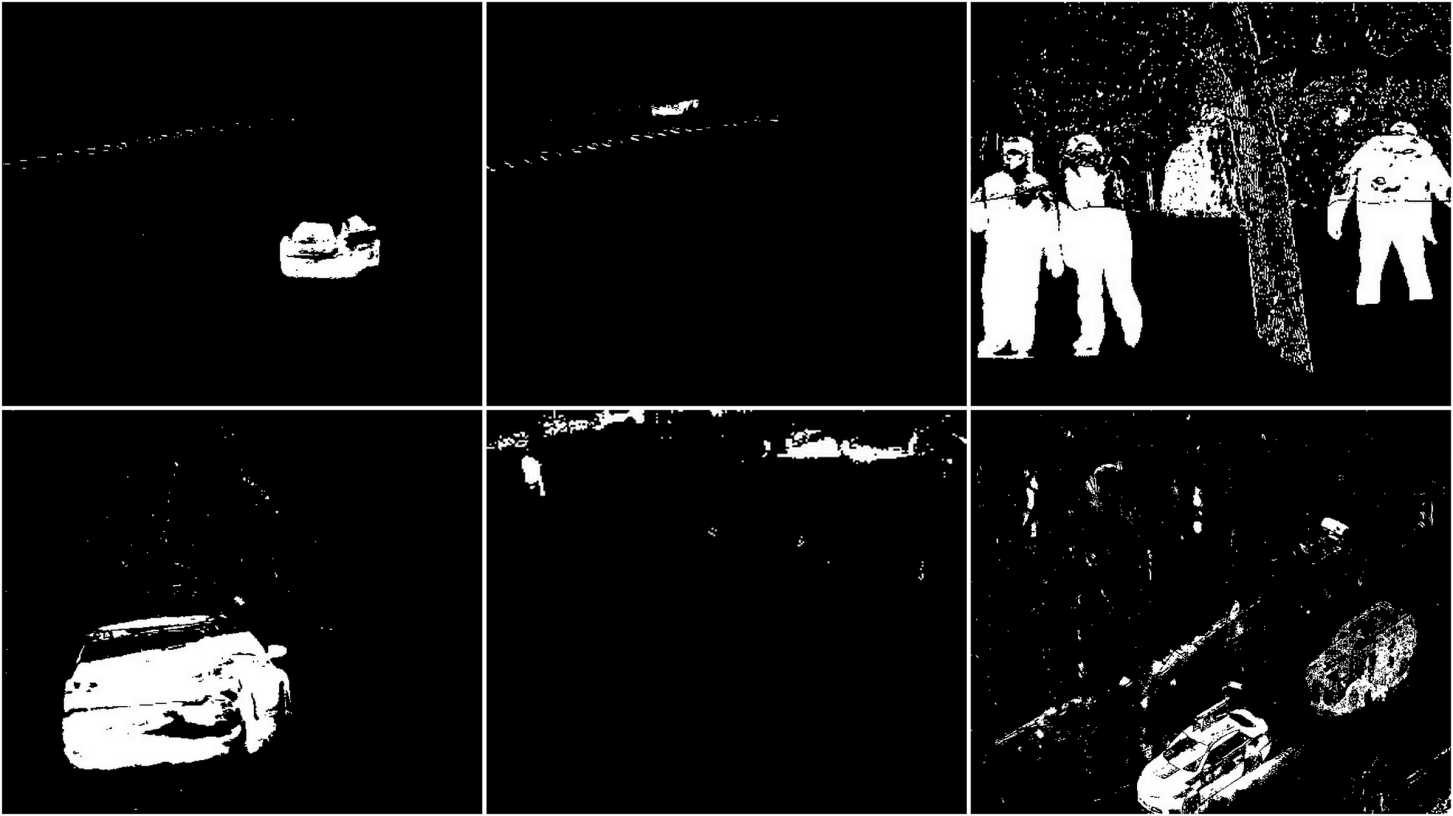
Results of the CNT algorithm during snowy weather.

**Figure 34:**
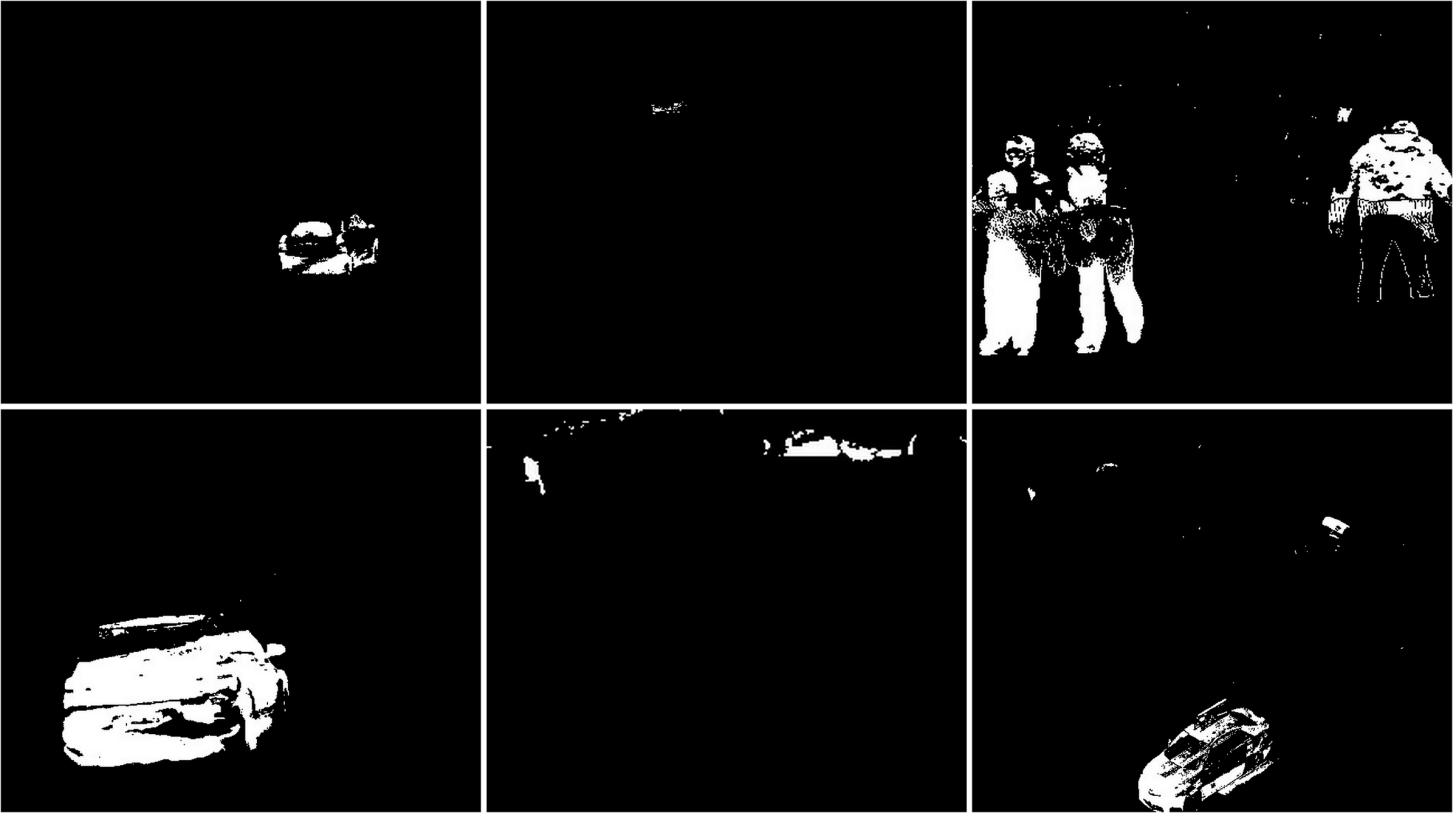
Results of the MOG algorithm during snowy weather.

**Figure 35:**
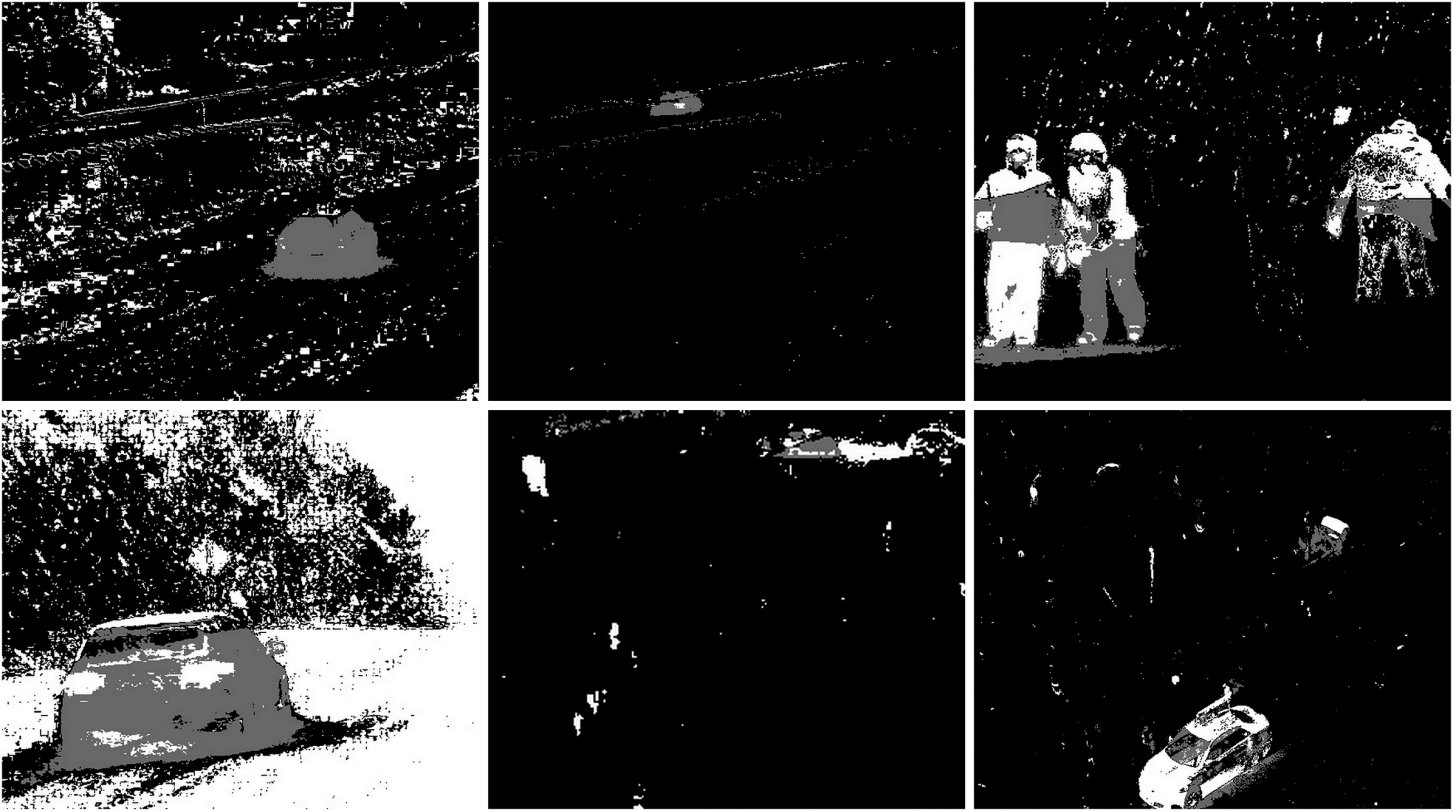
Results of the MOG2 algorithm during snowy weather.

**Figure 36:**
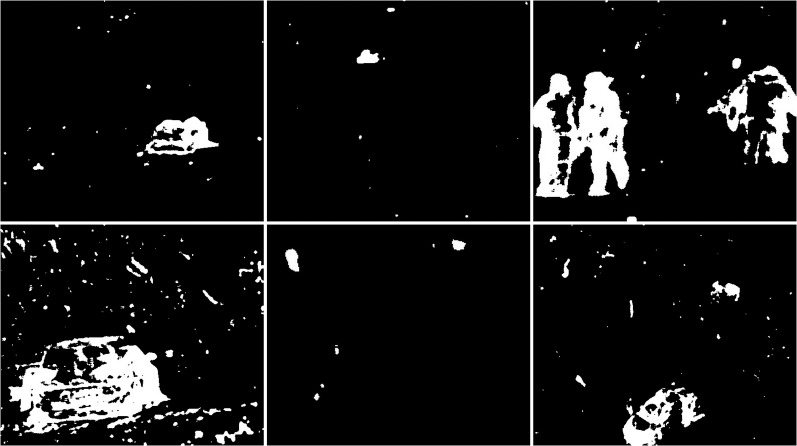
Results of the GMG algorithm during snowy weather.

**Figure 37:**
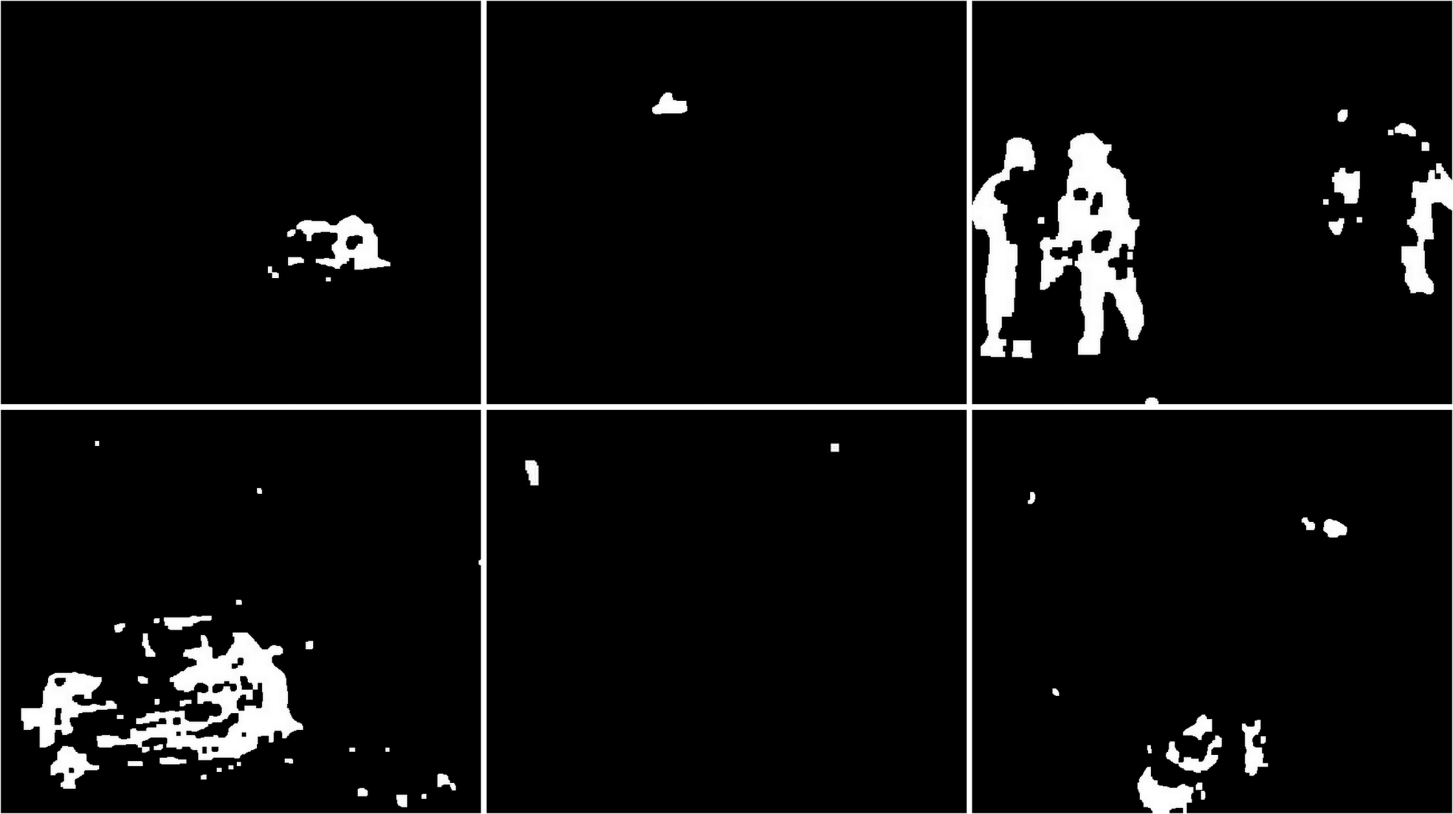
Results of updated GMG algorithm during snowy weather.

**Figure 38:**
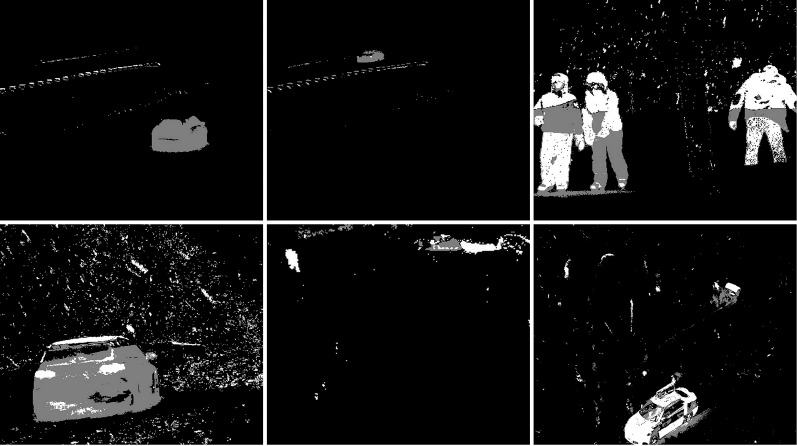
Results of the KNN algorithm during snowy weather.

**Figure 39:**
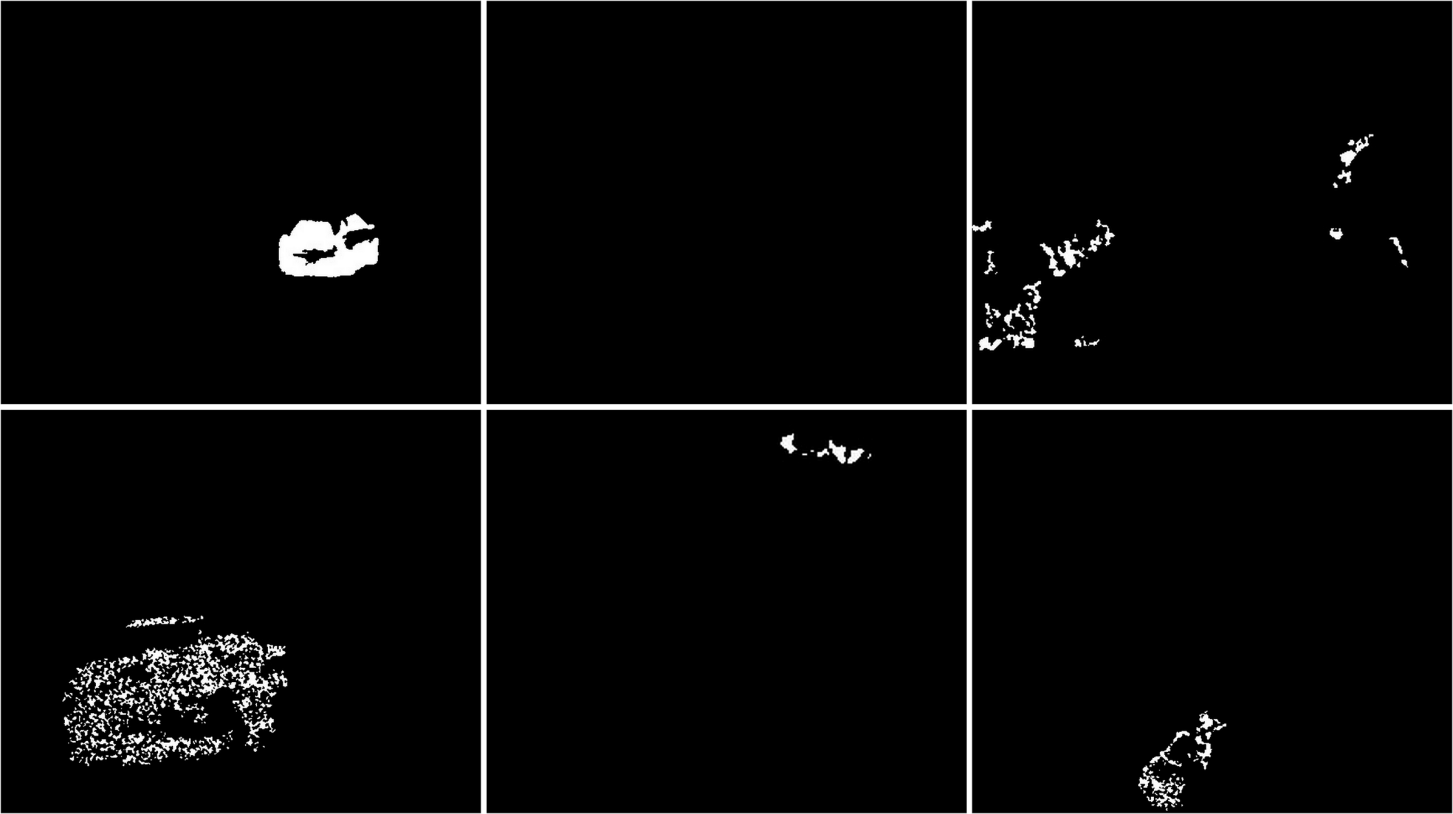
Results of the LSBP algorithm during snowy weather.

**Figure 40:**
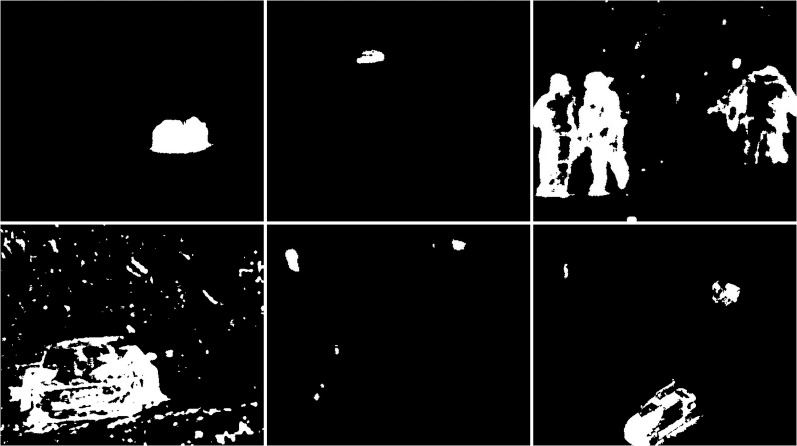
Results of the GSOC algorithm during snowy weather.

**Figure 41:**
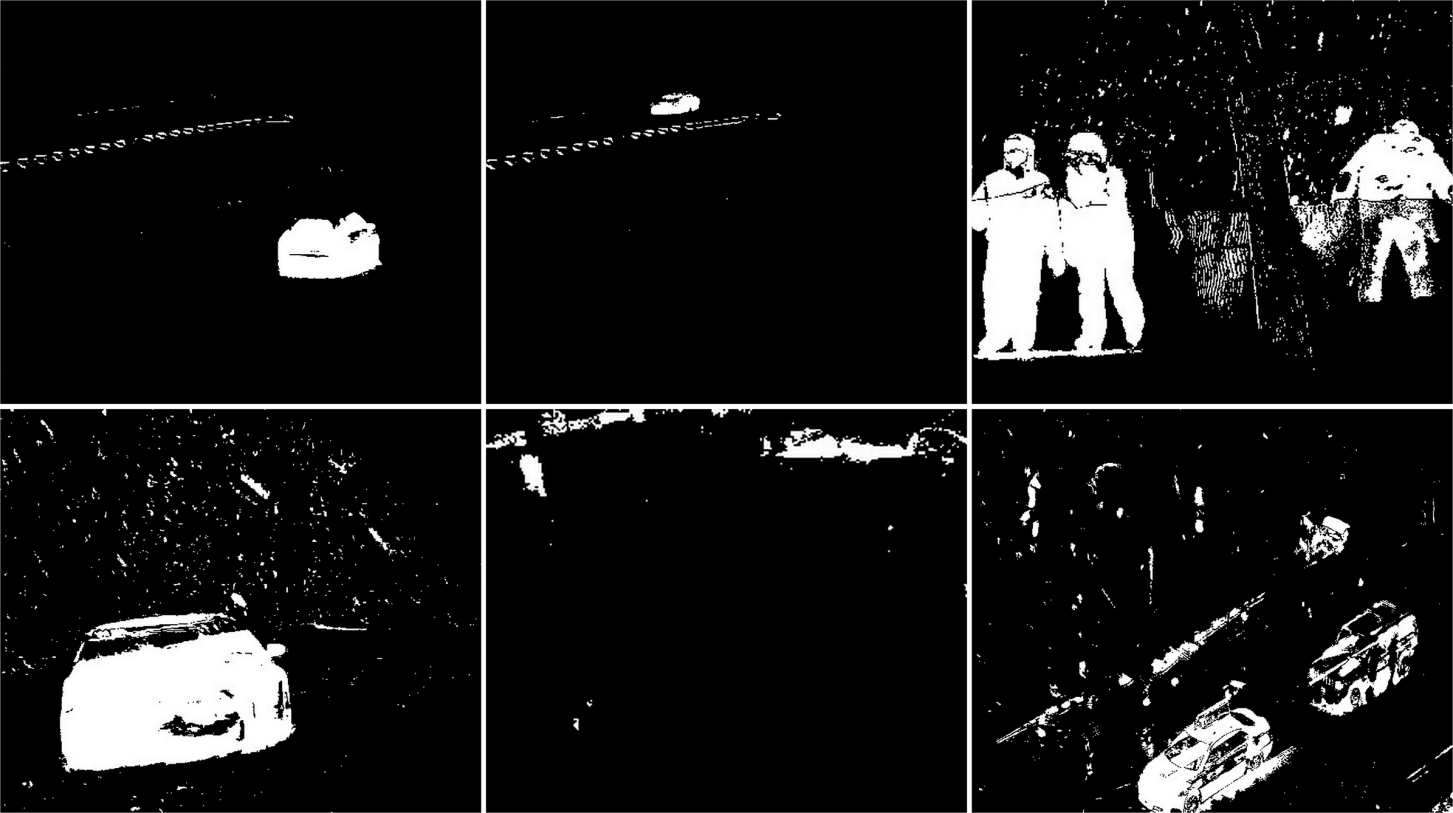
Results of the SOM algorithm during snowy weather.

#### Rain/wind

The rainy and windy weather condition images used in next step. For these conditions, frames containing cars and people were selected (see Fig. 42). The results of experiments on images during snowy weather are presented on Figs. 33–51.

**Figure 42:**
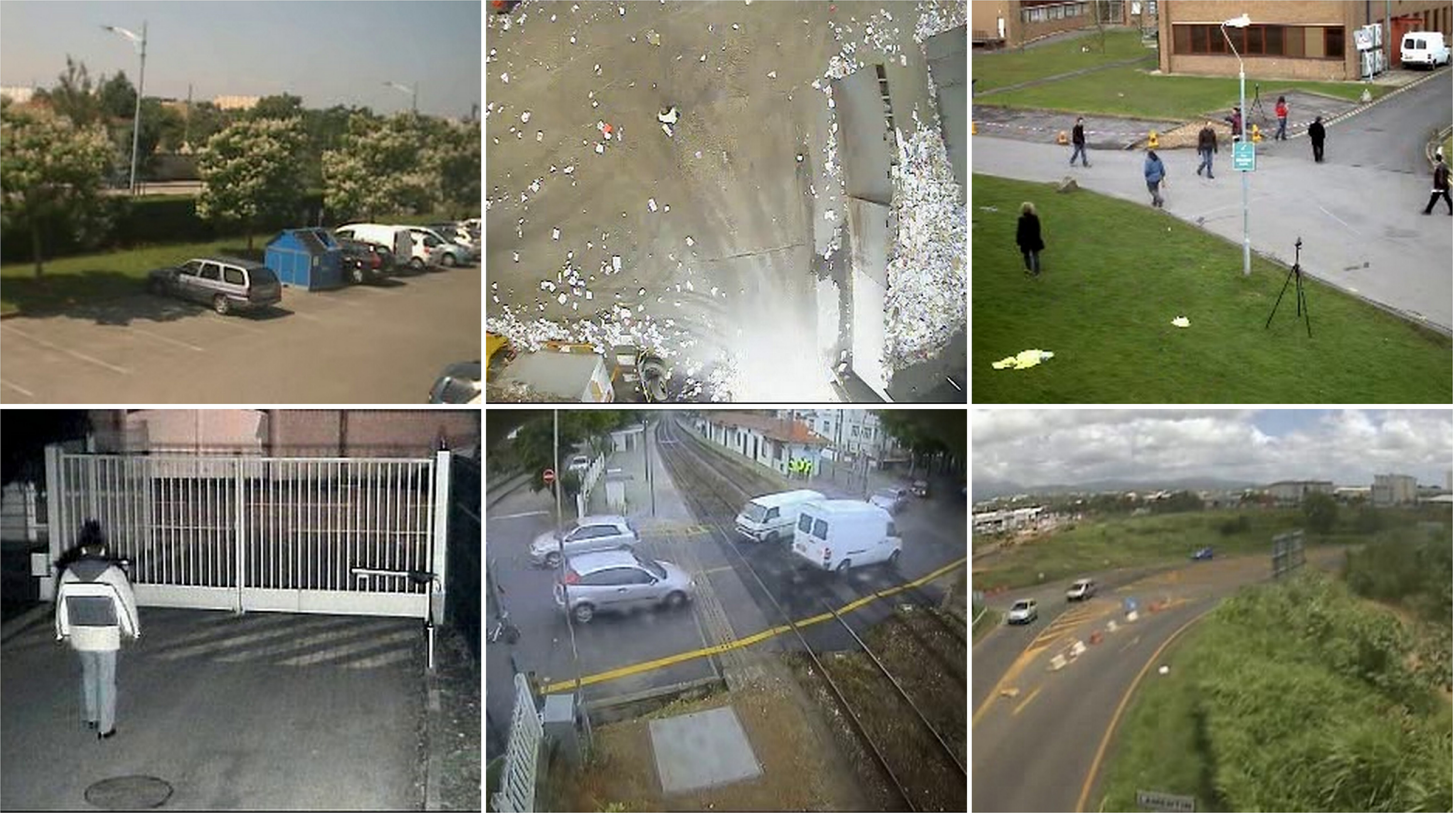
Original frame during rain.

**Figure 43:**
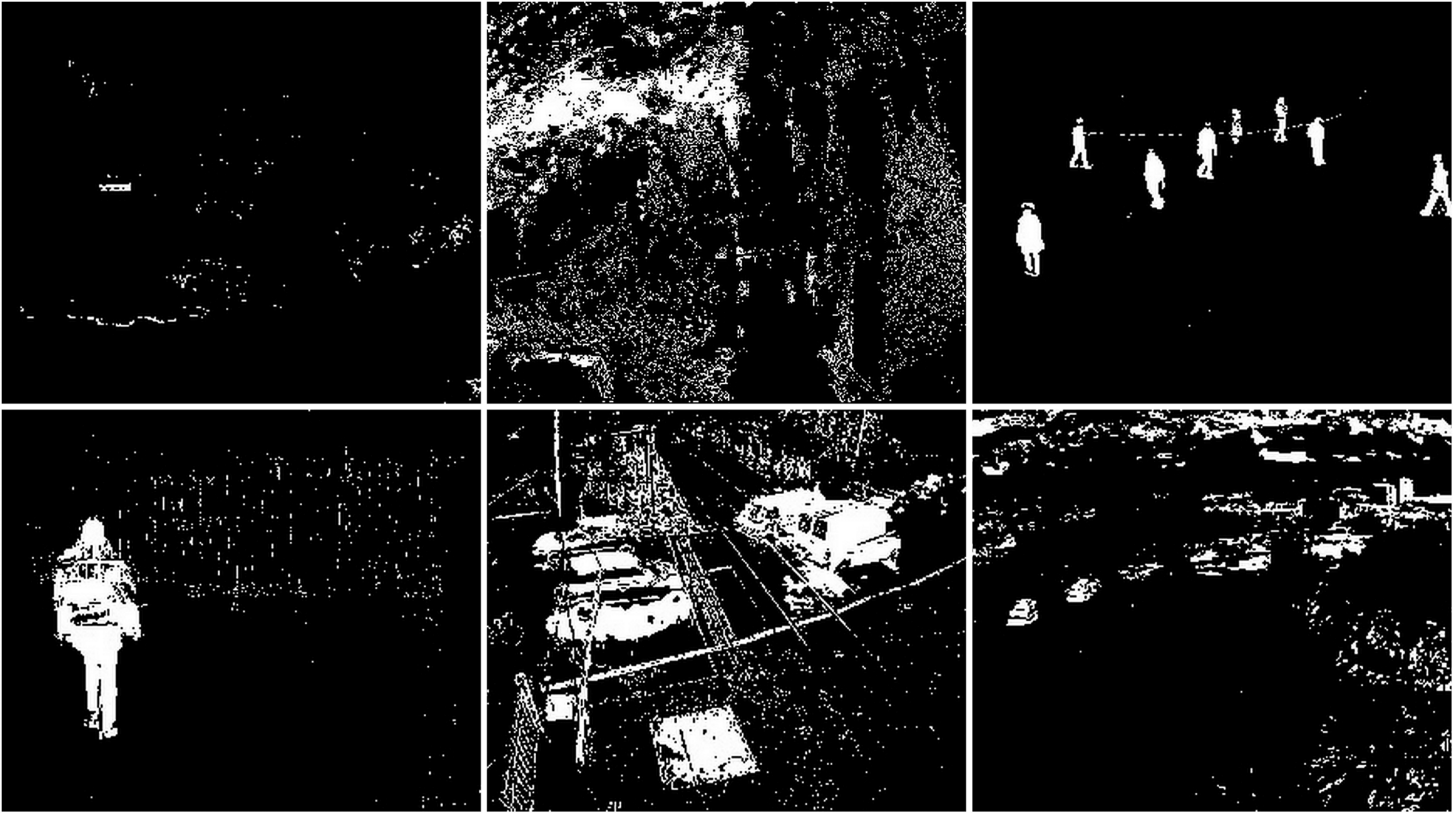
Results of the CNT algorithm during rainy weather.

**Figure 44:**
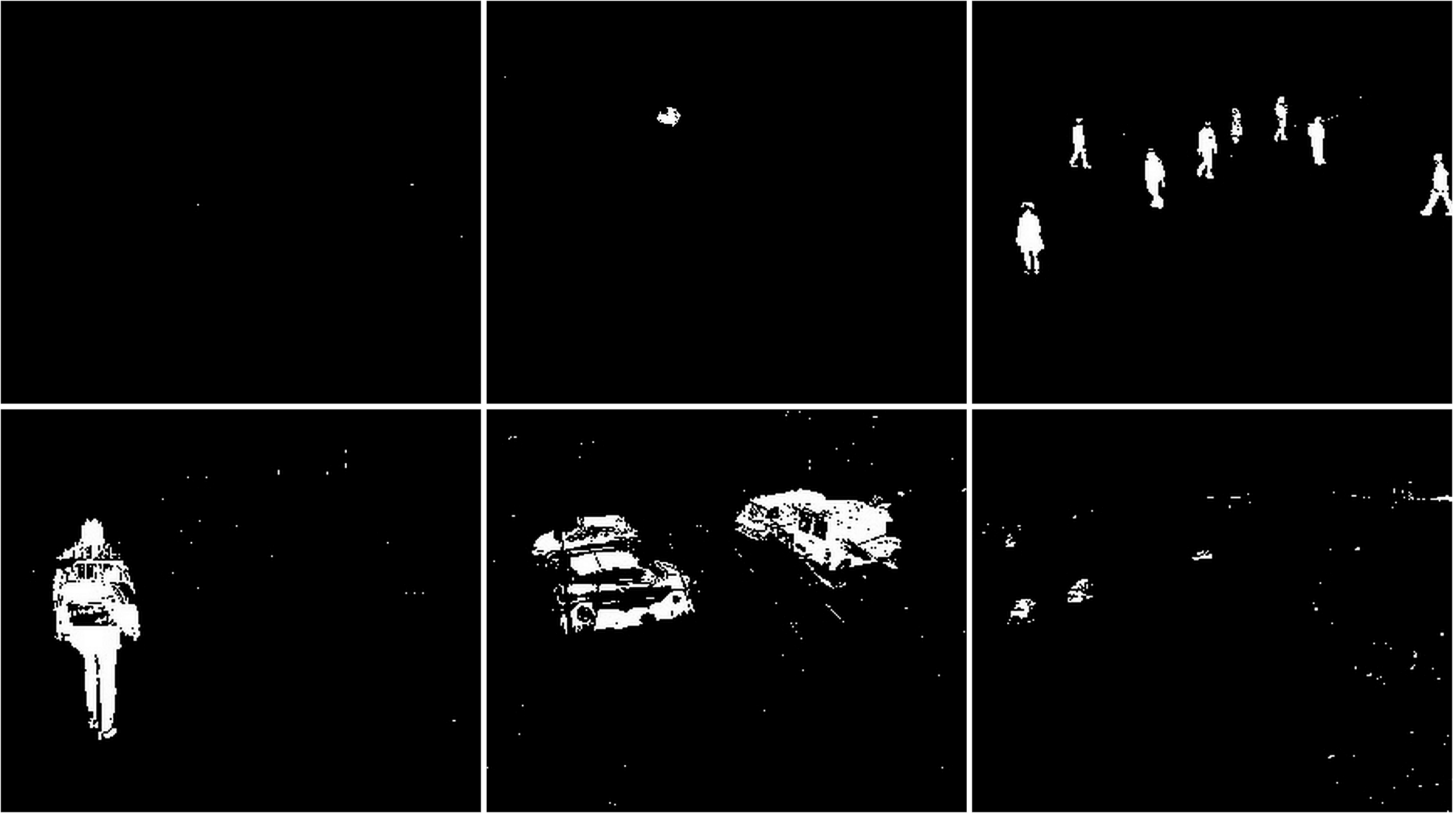
Results of the MOG algorithm during rainy weather.

**Figure 45:**
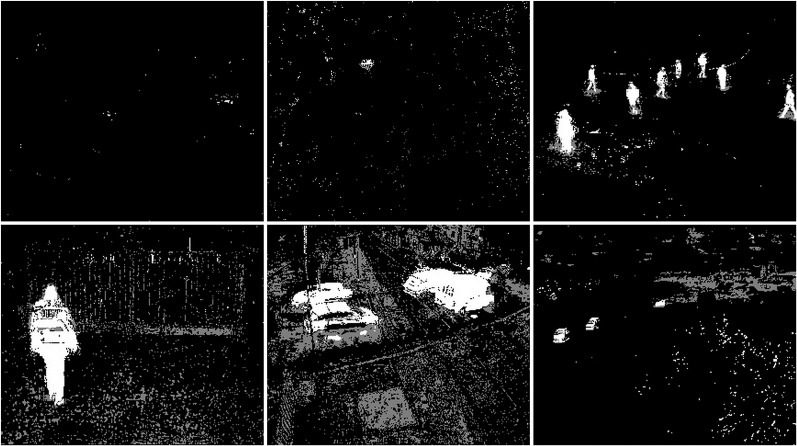
Results of the MOG2 algorithm during rainy weather.

**Figure 46:**
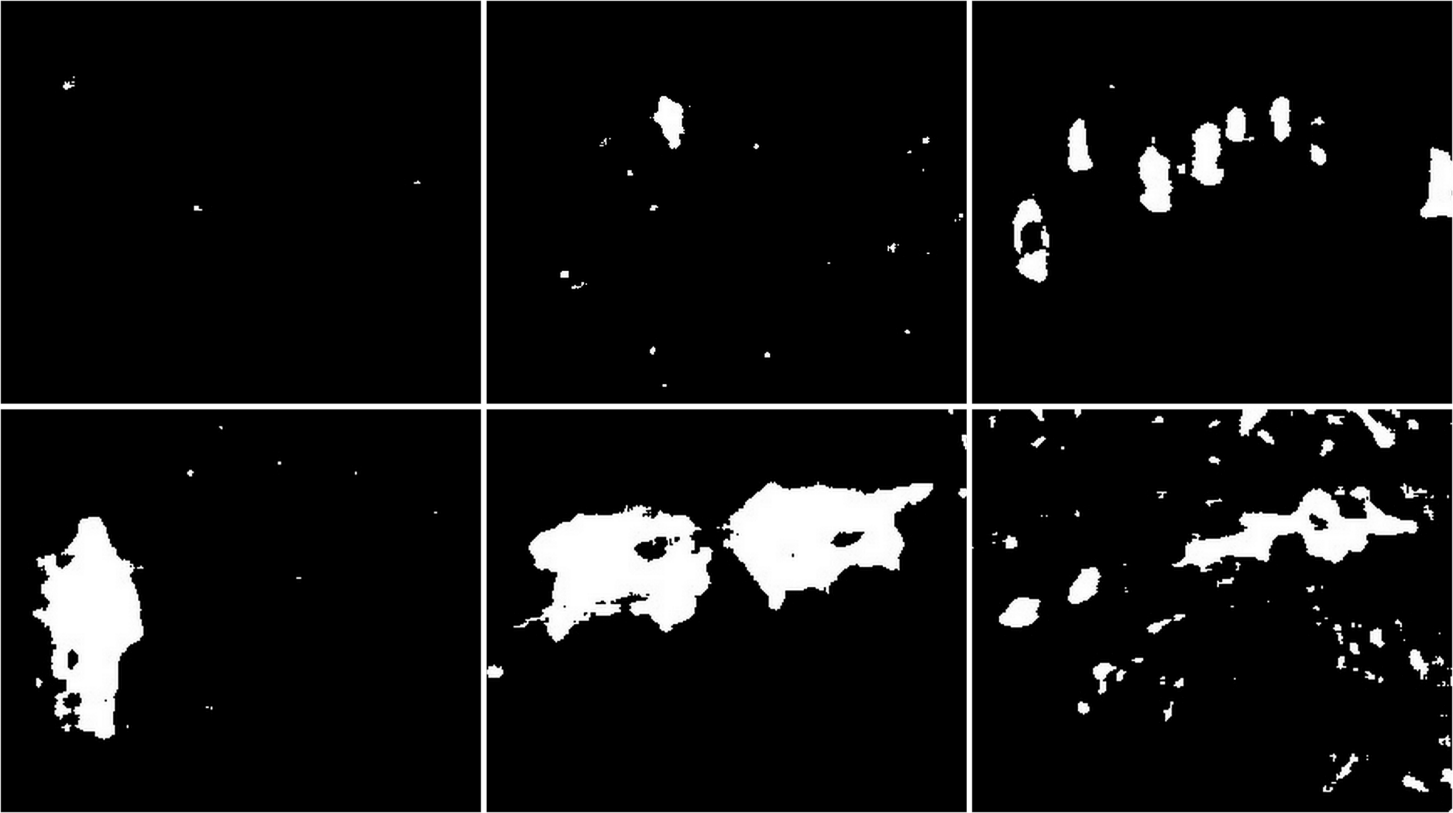
Results of the GMG algorithm during rainy weather.

**Figure 47:**
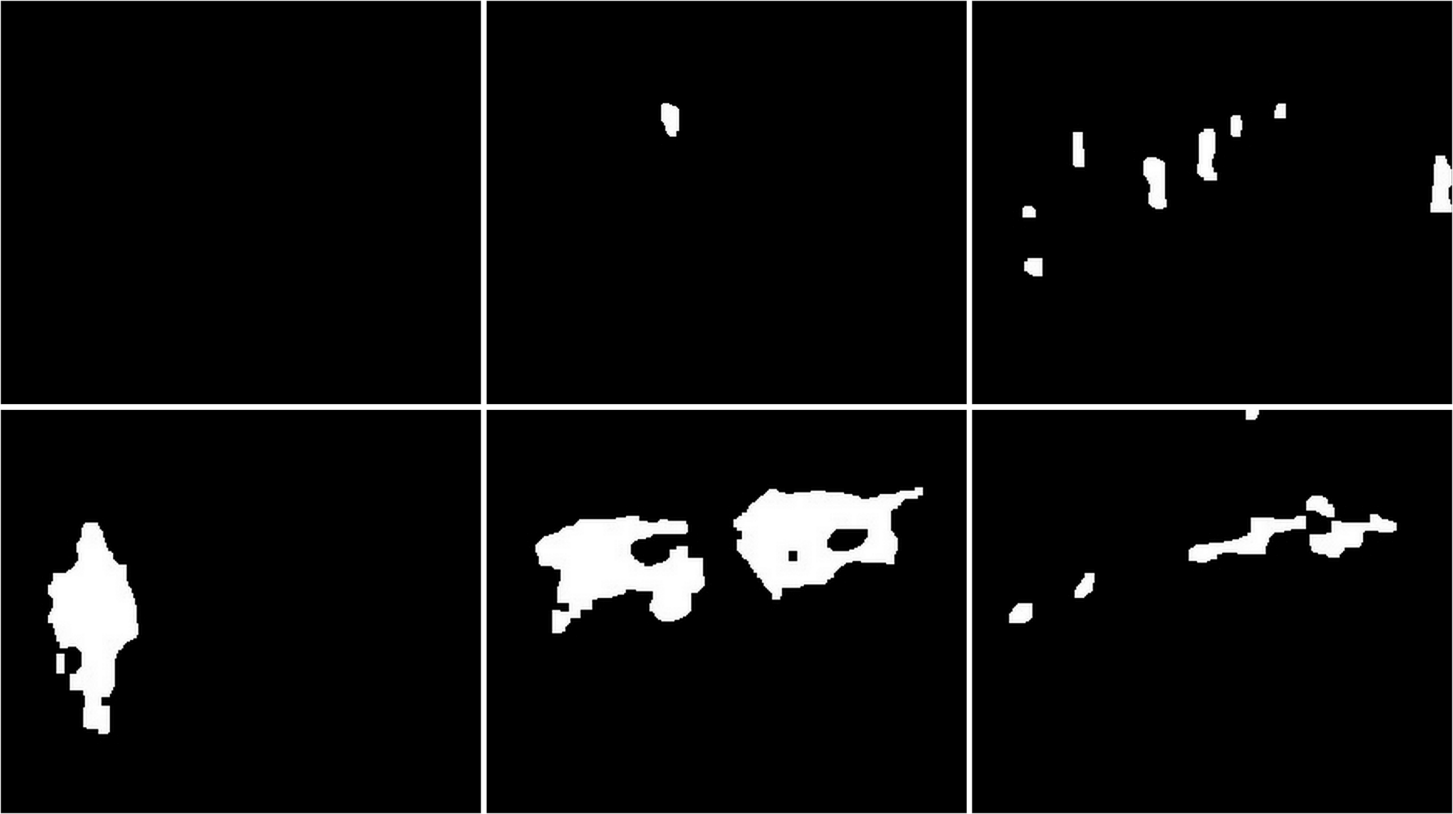
Results of updated GMG algorithm during rainy weather.

**Figure 48:**
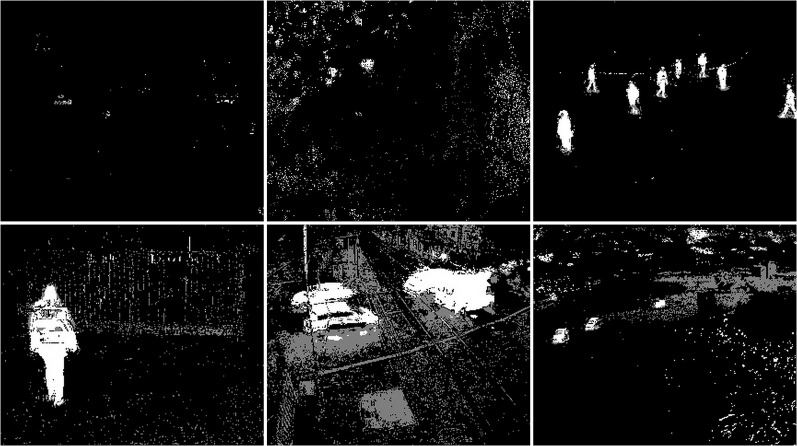
Results of the KNN algorithm during rainy weather.

**Figure 49:**
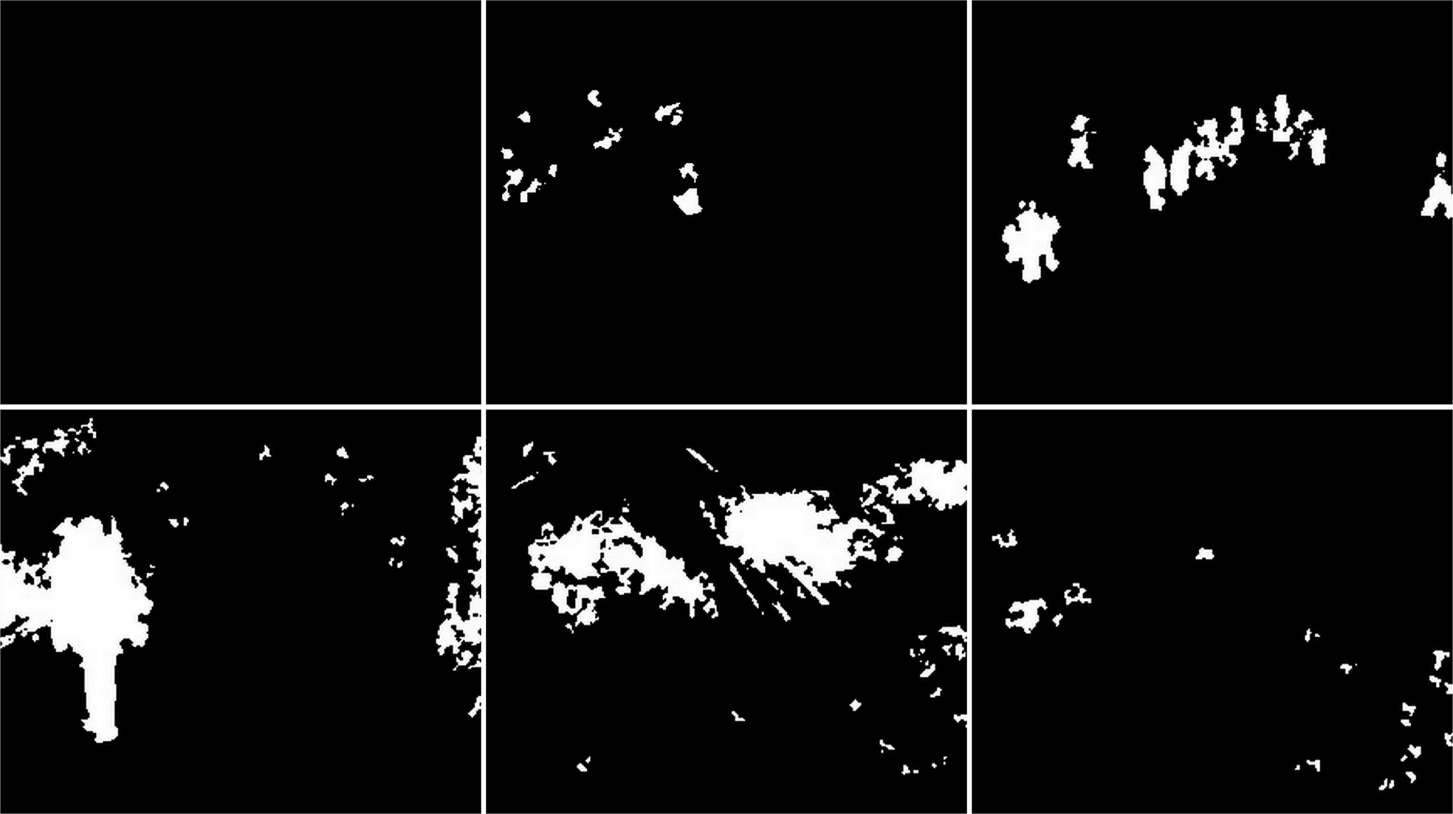
Results of the LSBP algorithm during rainy weather.

**Figure 50:**
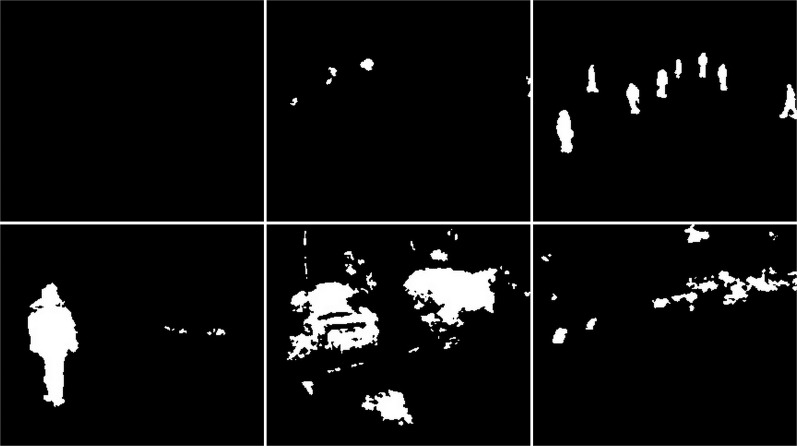
Results of the GSOC algorithm during rainy weather.

**Figure 51:**
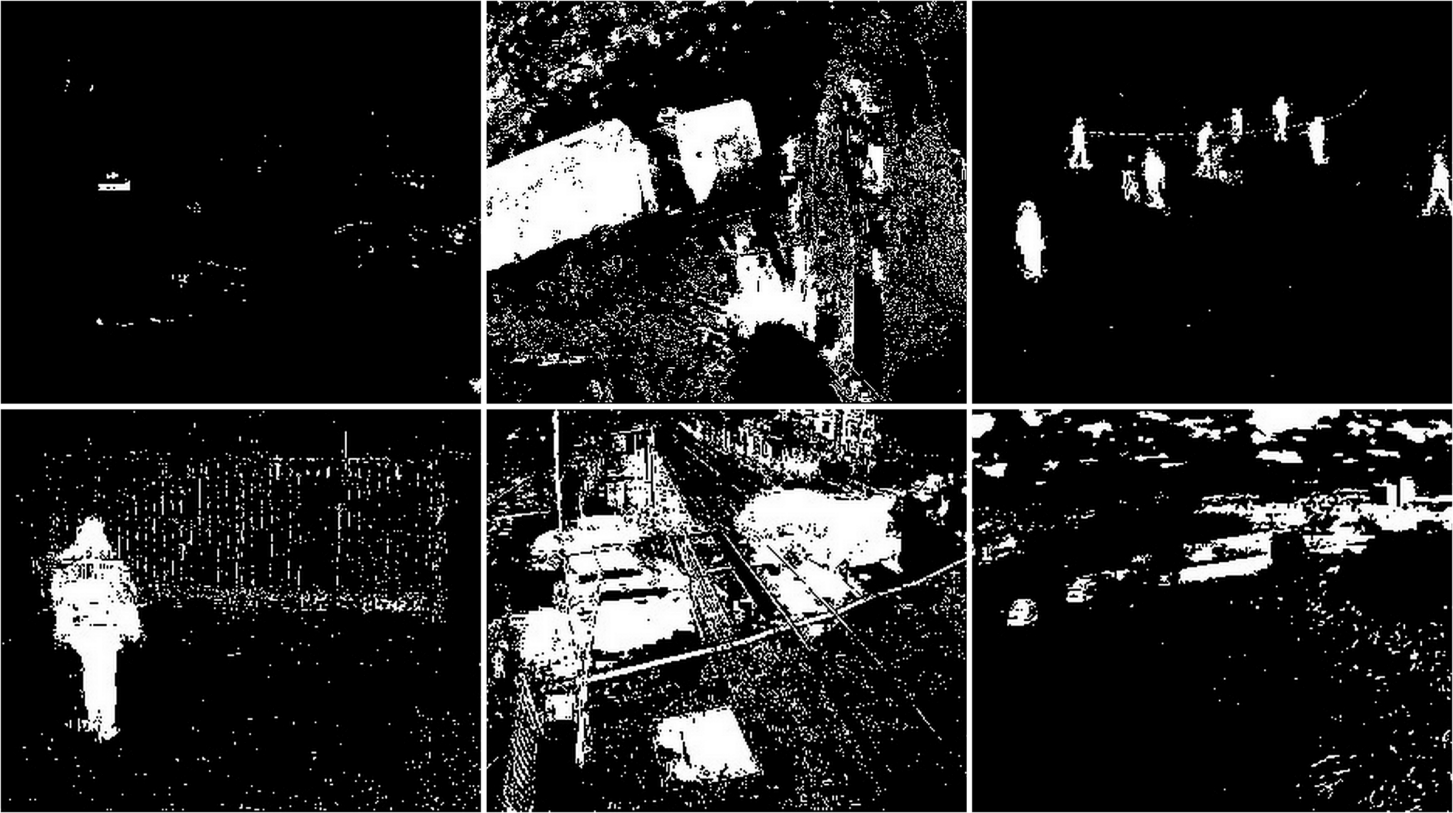
Results of the SOM algorithm during rainy weather.

## Results

Data on the amount of jamming present in the resulting images were collected and evaluated with three options in Table 1; the outcome was evaluated on the base visual evaluation. For the environment studied; LSBP and its improved version GSOC proved to be the most weather-resistant algorithms. The only noise obtained for these methods is a single cloud on a windy day. This is the middle image for the tests on a windy and cloudy day. The MOG2 and KNN algorithms recorded the most noise that was not the main moving object. These algorithms mostly detected the smallest changes in the images such as snowflakes or moving trees.

**Table 1:** The level of noises for each case studied.

	Weather condition		
Method	Snowfall	Windy and cloudy	Rainfall
CNT	m	m	s
MOG	s	none	none
MOG2	m	m	m
KNN	m	m	s
LSBP	s	s	none
GSOC	s	s	none
SOM	m	m	s
GMG	s	l	s
ourGMG	non	s	s

**Note:**

none, no noise; s, small; m, medium; l, large.

### Object mapping

The detail of detection of the main object, and other dynamic objects in the cases studied, was evaluated (see Table 2). In this duel also no single leader can be selected. MOG2 and KNN are very sensitive methods, which represent all dynamic objects very well. The CNT method also correctly represents the objects but at a lower level of detail. If the task of the method was only to make the outlines of the object visible, the GSOC algorithm would do well.

**Table 2:** Detail and shape mapping for the main moving object (% of object pixels).

	Weather condition			
Method	Snowfall	Windy and cloudy	Rainfall	Average
CNT	87	83	81	84
MOG	85	71	77	78
MOG2	95	87	87	90
KNN	86	81	94	87
LSBP	31	43	65	46
GSOC	98	47	96	80
SOM	90	81	94	88
GMG	92	43	92	92
ourGMG	93	89	91	91

Table 3 presents experiments results for own images with metrics IoU.

**Table 3:** IoU for our own images.

	Snow1	Snow2	Snow3	Wind1	Wind2	Wind3	Rain1	Rain2	Rain3	Avg.
CNT	0.29	0.08	0.09	0.33	0.05	0.02	0.50	0.58	0.61	0.28
KNN	0.25	0.11	0.13	0.33	0.04	0.04	0.75	0.56	0.63	0.32
MOG	0.51	0.24	0.17	0.62	0.23	0.15	0.44	0.57	0.62	0.39
MOG2	0.20	0.11	0.12	0.29	0.02	0.03	0.65	0.39	0.47	0.25
GMG	0.59	0.52	0.49	0.16	0.07	0.14	0.58	0.55	0.67	0.42
ourGMG	0.79	0.53	0.29	0.64	0.46	0.07	0.62	0.62	0.74	0.53
GSOC	0.73	0.48	0.71	0.68	0.22	0.01	0.71	0.61	0.63	0.53
LSBP	0.42	0.42	0.00	0.54	0.32	0.01	0.34	0.09	0.28	0.27
SOM	0.62	0.28	0.36	0.62	0.08	0.17	0.74	0.64	0.71	0.47

Tables 4 and 5 presents experiments results for own images with metrics IoU. The results obtained in the second stage of the experiment, where the dataset was from public resources, coincide to a greater extent with the data from the first stage. We see the difference for some images with snowy weather, in this case the GSOC and SOM algorithms turned out to be the best. However, the better result comes with a longer computation time, for GSOC it is almost five times longer than for ourGMG, and for SOM twice as long.

**Table 4:** IoU for snowy weather from a public database.

	Snow1	Snow2	Snow3	Snow4	Snow5	Snow6	Avg.
CNT	0.63	0.21	0.48	0.75	0.11	0.26	0.41
KNN	0.52	0.20	0.62	0.36	0.11	0.46	0.38
MOG	0.44	0.20	0.54	0.74	0.19	0.50	0.44
MOG2	0.10	0.13	0.53	0.15	0.09	0.49	0.25
GMG	0.51	0.45	0.57	0.39	0.12	0.48	0.42
ourGMG	0.49	0.54	0.58	0.43	0.27	0.40	0.45
GSOC	0.71	0.73	0.72	0.74	0.23	0.55	0.61
LSBP	0.76	0.00	0.11	0.44	0.26	0.19	0.29
SOM	0.73	0.40	0.71	0.74	0.18	0.36	0.52

**Table 5:** IoU for rainy weather from a public database.

	Video1	Video2	Video3	Video4	Video5	Video6	Avg.
CNT	0.00	0.01	0.46	0.3	0.2	0.03	0.17
KNN	0.00	0.01	0.42	0.17	0.18	0.03	0.14
MOG	0.00	0.43	0.51	0.63	0.5	0.13	0.37
MOG2	0.00	0.02	0.26	0.13	0.17	0.03	0.10
GMG	0.00	0.2	0.39	0.63	0.57	0.05	0.31
ourGMG	1.00	0.58	0.41	0.77	0.62	0.14	0.59
GSOC	1.00	0.25	0.54	0.76	0.46	0.1	0.52
LSBP	1.00	0.09	0.36	0.32	0.42	0.16	0.39
SOM	0.00	0.01	0.62	0.52	0.27	0.05	0.25

### Computation time

The computation time is the time required to complete the computation process. It is closely related to the complexity of a given algorithm. Less complexity translates into faster algorithm performance, which is critical for real-time systems (see Table 6). The hardware used in experiment: Operating system Ubuntu 20.04, 16GB RAM, 4 core i7-10870H @ 2.2 GHz.

**Table 6:** Computation time of algorithms (s).

Method	Min	Max	Avg.
CNT	0.001	0.015	0.007
MOG	0.006	0.024	0.011
MOG2	0.002	0.012	0.082
KNN	0.002	0.025	0.013
LSBP	0.025	0.144	0.086
GSOC	0.011	0.177	0.095
SOM	0.032	0.059	0.038
GMG	0.009	0.083	0.046
ourGMG	0.009	0.033	0.021

## Conclusions

This article compares the popular methods for background clipping public available. Implicit values were assumed for all algorithms. Their results were examined during weather conditions that could potentially generate detection interference. The study allowed for the identification of the best algorithms for two categories.

The first category consists of algorithms with the lowest susceptibility to interference caused by changing weather conditions. The leader of this category was the GSOC method. The only case with unsatisfactory result is the third image on a windy day. The threshold removed small birds, considering them as noise. Other than that, the algorithm showed the main objects correctly. It reproduced their shape well.

The second category is the methods most sensitive to changes in the image. Here the slight leader turned out to be the MOG2 algorithm. In the results it showed the most detailed objects. However, it was very susceptible to interference caused by weather conditions. Its rivals worth mentioning are KNN and CNT algorithms. KNN gave very comparable results, however in a few cases it was slightly worse, which is why it was allocated to second place. The third method worth considering for detail is CNT, which should be the most efficient method on weak hardware. It would be interesting to compare the performance of these three algorithms on, for example, a Raspberry Pi and see what load they generate.

As can be seen from the obtained results, the proposed algorithm is characterised by similar effectiveness as the best (GMG, MOG2) and better than the others. The same applies to computing time which is small enough to be used in data processing on an ongoing basis. In addition, our algorithm provides a lower noise level than the original one.

Summarizing the research results and their analysis, the article presents a new modified background segmentation method characterized by good efficiency and a relatively short calculation time. These features qualify this algorithm for real-time use in an autonomous car.

## Supplemental Information

10.7717/peerj-cs.962/supp-1 Supplemental Information 1 Code.

10.7717/peerj-cs.962/supp-2 Supplemental Information 2 Raw data. Input images

## Additional Information and Declarations

### Data Availability

The following information was supplied regarding data availability: The raw data and code are available in the Supplemental Files.
