# Supplementary figures and images for: Background segmentation in difficult weather conditions
Source: PeerJ Comput Sci. 2022 May 13;8:e962. doi: 10.7717/peerj-cs.962 (PMC9137877; doi:10.7717/peerj-cs.962)

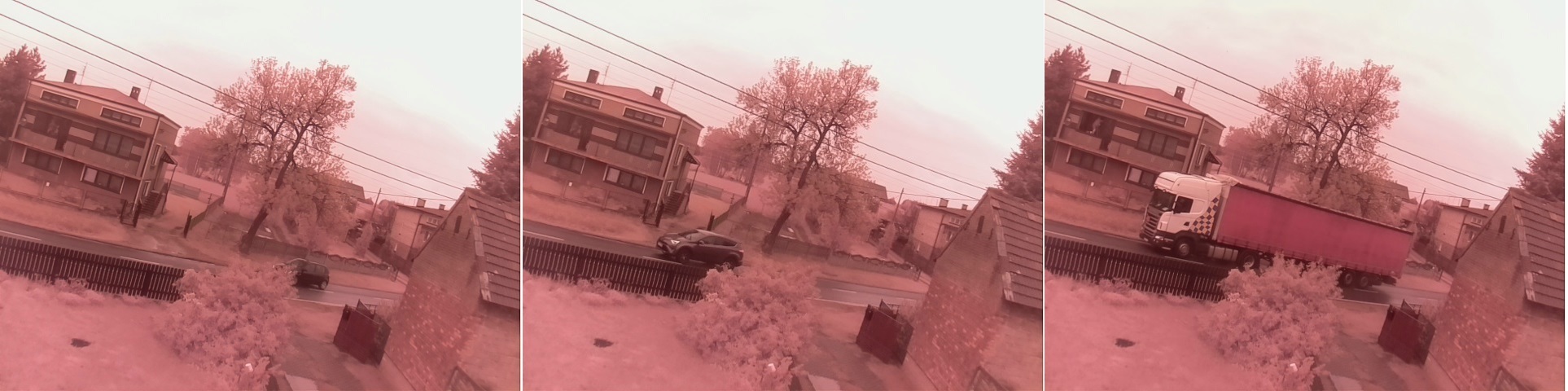

Supplement: Supplemental Information 2 — Input images [file peerj-cs-08-962-s002.zip › frame_rain.jpg]

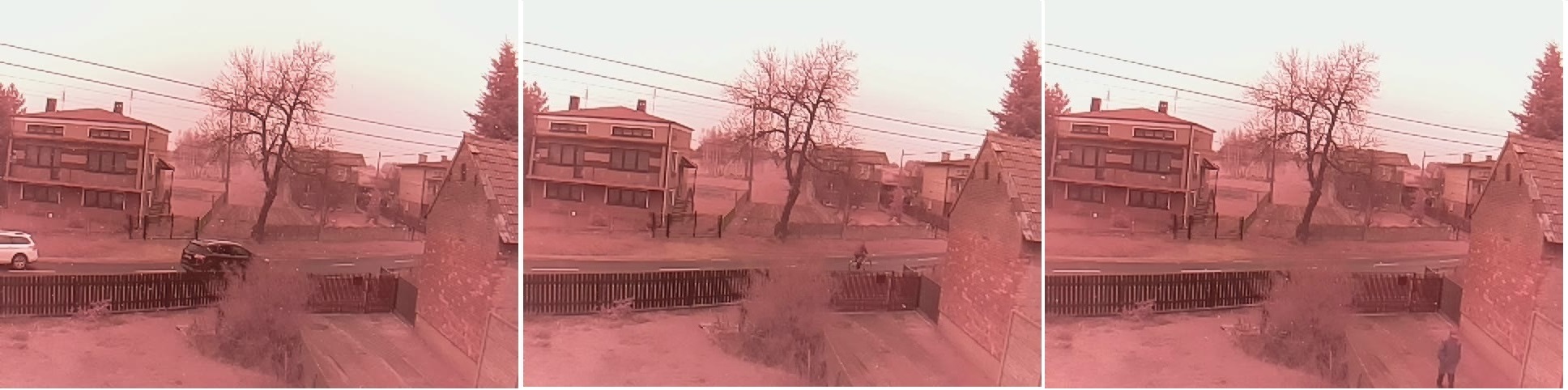

Supplement: Supplemental Information 2 — Input images [file peerj-cs-08-962-s002.zip › frame_snow.jpg]

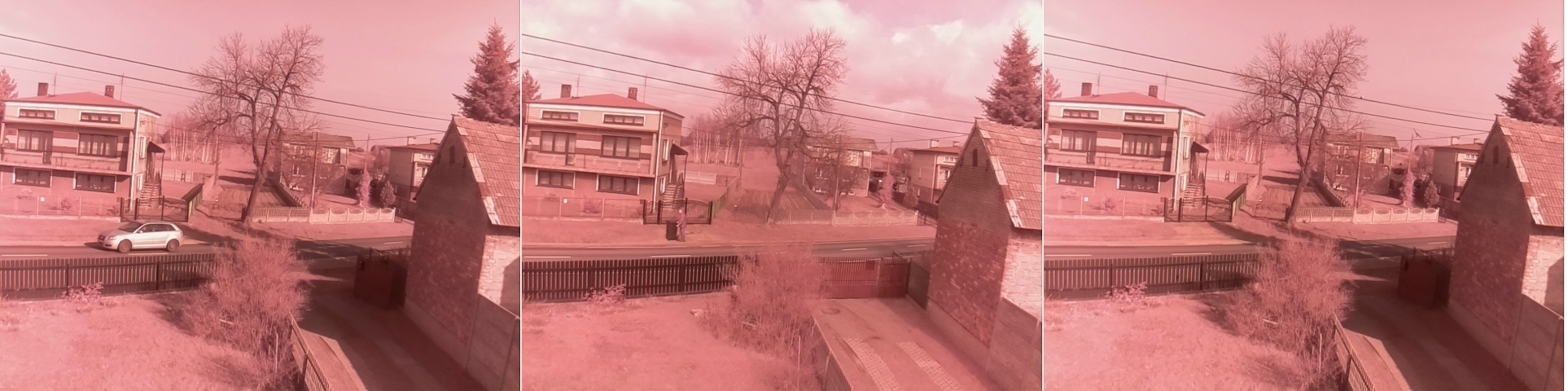

Supplement: Supplemental Information 2 — Input images [file peerj-cs-08-962-s002.zip › frame_wind.JPG]
